# The CUL3-SPOP-DAXX axis is a novel regulator of *VEGFR2* expression in vascular endothelial cells

**DOI:** 10.1038/srep42845

**Published:** 2017-02-20

**Authors:** Tomohisa Sakaue, Iori Sakakibara, Takahiro Uesugi, Ayako Fujisaki, Koh-ichi Nakashiro, Hiroyuki Hamakawa, Eiji Kubota, Takashi Joh, Yu-ki Imai, Hironori Izutani, Shigeki Higashiyama

**Affiliations:** 1 Division of Cell Growth and Tumor Regulation, Proteo-Science Center (PROS), Ehime University, Toon, Ehime, Japan; 2 Department of Cardiovascular and Thoracic Surgery, Ehime University Graduate School of Medicine, Ehime, Japan; 3 Department of Integrative Pathophysiology, Proteo-Science Center (PROS), Ehime University, Toon, Ehime, Japan; 4 Department of Biochemistry and Molecular Genetics, Ehime University Graduate School of Medicine, Toon, Ehime, Japan; 5 Department of Oral and Maxillofacial Surgery, Ehime University, Graduate School of Medicine, Ehime University, Toon, Ehime, Japan; 6 Department of Gastroenterology and Metabolism, Nagoya City University Graduate School of Medical Sciences, Nagoya, Japan

## Abstract

Vascular endothelial cell growth factor receptor 2 (VEGFR2) is an essential receptor for the homeostasis of endothelial cells. In this study, we showed that NEDD8-conjugated Cullin3 (CUL3)-based ubiquitin E3 (UbE3) ligase plays a crucial role in *VEGFR2* mRNA expression. Human umbilical vein endothelial cells treated with MLN4924, an inhibitor of NEDD8-activating enzyme, or with CUL3 siRNA drastically lost their response to VEGF due to the intense decrease in *VEGFR2* expression. Moreover, speckle-type POZ protein (SPOP) and death-domain associated protein (DAXX) were involved in the CUL3 UbE3 ligase complex as a substrate adaptor and a substrate, respectively. Knockdown of SPOP and CUL3 led to the upregulation of DAXX protein and downregulation of VEGFR2 levels. These levels were inversely correlated with one another. In addition, simultaneous knockdown of SPOP and DAXX completely reversed the downregulation of VEGFR2 levels. Moreover, the CUL3-SPOP-DAXX axis had the same effects on *NOTCH1, DLL4* and *NRP1* expression. Taken together, these findings suggest that the CUL3-SPOP-DAXX axis plays a very important role in endothelial cell function by targeting key angiogenic regulators.

New blood vessel formation, termed angiogenesis, is an essential process in normal physiology, including tissue development and wound healing, as well as in many pathological conditions such as cancer and diabetes, among other^1^,^2^. Endothelial cells play a central role in angiogenesis, and the major driving force for endothelial cell activation is signaling through vascular endothelial growth factors (VEGFs) and their receptors (VEGFRs). Among the VEGF-VEGFR signaling pathways, the VEGF-VEGFR2 axis is the most prominent pathway in angiogenesis. Therefore, targeting this signaling pathway is one of the most promising anti-angiogenic strategies. To establish angiogenic therapies, detailed studies of the molecular mechanisms underlying angiogenesis have been conducted. For example, such studies have led to the development of therapeutic agents such as Avastin and their clinical application. However, the clinical outcomes of angiogenic therapies have not been satisfactory, indicating the need for additional approaches.

The VEGF-VEGFR2 signaling axis remains an important therapeutic target. Most previous studies have focused on the transcriptional and translational regulation of VEGF and VEGFR2. Recently, regulation via post-transcriptional and post-translational mechanisms has gained attention in studies of angiogenesis. Thus, microRNAs such as *microRNA-16* have been reported to target *VEGFR2* mRNA at the post-transcriptional level^3^,^4^, and SCFβ-TRCP has been found to ubiquitinate and degrade VEGFR2 protein^5^. Furthermore, neddylation^6^, which involves the conjugation of the ubiquitin-like protein NEDD8 to its target protein, is a crucial post-translational modification in addition to ubiquitination. Neddylation is reportedly required for angiogenic regulation. Importantly, MLN4924, an inhibitor of NEDD8-activating enzyme (NAE), blocks angiogenesis in various models *in vitro* and *in vivo*^7^. MLN4924 is known to inhibit Cullin (CUL)-RING UbE3 ligase activity, which requires CUL protein modification by NEDD8. MLN4924 has also been reported to suppress the survival of several tumors as well as their growth and metastasis. MLN4924 is currently in Phase I clinical trials for the treatment of cancer^8^,^9^,^10^,^11^,^12^.

The *CUL* gene family in humans comprises eight members (*CUL1, CUL2, CUL3, CUL4A, CUL4B, CUL5, CUL7* and *PARC*). The amino acid sequences of these members are highly homologous^13^. Previous studies have indicated that CULs regulate angiogenesis. For example, CUL2 positively regulates hypoxia-induced angiogenesis by degrading von Hippel-Lindau tumor suppressor (VHL), which is a UbE3 ligase for hypoxia-inducible factor (HIF-1)^14^. CUL5 regulates angiogenesis through MAPK phosphorylation and actin polymerization^15^. Furthermore, our recent studies unveiled the crosstalk mechanisms between VEGF and Notch signaling via CUL3-mediated protein degradation pathways. VEGF-A induces the stabilization of *BAZF* (also known as B-cell chronic lymphocytic leukemia/lymphoma 6 member B, *BCL6B*) mRNA, resulting in an increase in BAZF protein that mediates the poly-ubiquitination and degradation of RBP-Jκ/CBF-1, a key transcriptional regulator of Notch signaling, with CUL3 UbE3 ligase^16^,^17^.

In this study, we found that NEDD8-conjugated CUL3 was essential for the activation of VEGF signal transduction by inducing *VEGFR2* expression. We also report that activated CUL3 positively regulated angiogenesis by inducing the expression of *VEGFR2* as well as *NRP1, NOTCH1* and *DLL4.*

## Results

### MLN4924 suppressed *VEGFR2* expression

**Figure 1:**
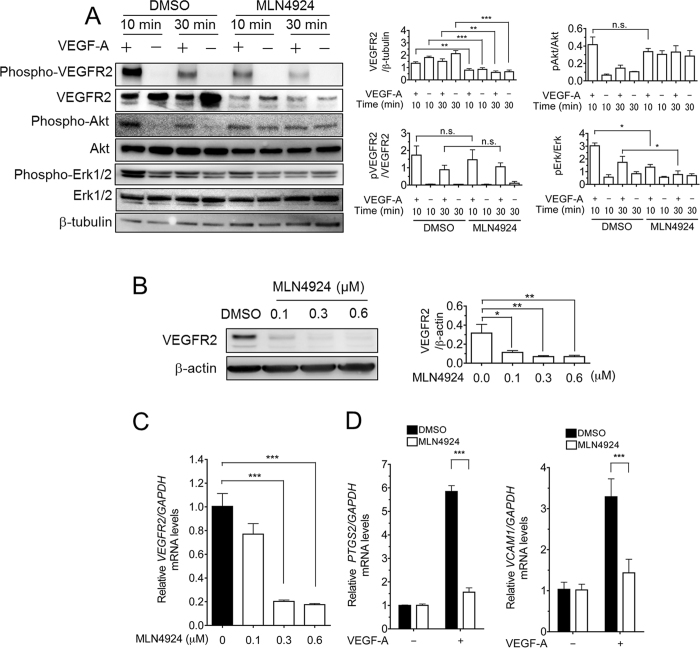
VEGF-A-induced endothelial activation was required for NAE activity. (**A**) MLN4924 abrogated VEGF-A-stimulated cell activation of VEGFR2, Akt and Erk1/2. HUVECs were pretreated with MLN4924 (0.3 μM) or DMSO for 72 h. After serum starvation for 12 h, the HUVECs were then stimulated with 50 ng/mL VEGF-A for 10 or 30 min. Total or phosphorylated forms of VEGFR2, Akt and Erk1/2 were detected by immunoblotting. β-tubulin was used as an internal control. The VEGFR2 protein levels and phosphorylated levels of VEGFR2, Akt and Erk were quantified using ImageJ software (*right panels*). *, p < 0.05. (**B**) HUVECs were treated with 0.1, 0.3 or 0.6 μM MLN4924 for 72 h. VEGFR2 protein levels in HUVECs were analyzed by Western blotting using an anti-VEGFR2 antibody. The relative intensity of each band was statistically analyzed using ImageJ software and normalized relative to β-actin (loading control). (**C**) *VEGFR2* mRNA in HUVECs treated with 0.1, 0.3 or 0.6 μM MLN4924 for 72 h was measured by qRT-PCR. *VEGFR2* mRNA levels were normalized to *GAPDH* mRNA. ***p < 0.001. (**D**) HUVECs were pretreated with DMSO or 0.3 μM MLN4924 for 72 h. The cells were stimulated with VEGF-A (50 ng/mL) for 2 h. *PTGS2 (left panel*) and *VCAM1 (right panel*) mRNA levels were then measured by qRT-PCR. The relative target mRNA levels were normalized to *GAPDH* mRNA. ***p < 0.001. The experiments were performed independently 3 times.

It has been previously been shown that MLN4924, an inhibitor of NEDD8-activating E1 enzyme, suppresses VEGF-A-induced angiogenesis *in vivo* and *in vitro*^7^. Using an aortic ring assay, we also found that VEGF-A-induced angiogenesis was strongly inhibited by MLN4924 (data not shown). To reveal the molecular mechanisms underlying this phenomenon, we first analyzed the effects of MLN4924 on the VEGF signal transduction pathway by examining the activation levels of essential kinases, including VEGFR2, Akt and Erk1/2, in cultured HUVECs. HUVECs were treated with MLN4924 (0.3 μM) or Dimethyl sulfoxide (DMSO) for 72 h before VEGF-A stimulation. Without MLN4924, Erk1/2 and VEGFR2 were strongly phosphorylated by VEGF-A stimulation after 10 min, and then the levels gradually decreased. By contrast, cells pretreated with MLN4924 displayed almost no upregulation of phosphorylation of VEGFR2 (Fig. 1A), whereas the efficiency, phosphorylation per receptor content, of VEGFR2 phosphorylation remained unchanged. VEGF-A also induced phosphorylation of Akt in control cells from 10 to 30 min. In MLN4924-treated cells, VEGF-induced phosphorylation of Akt was scarcely observed, whereas high basal levels of Akt phosphorylation were observed compared with the controls (Fig. 1A). Unexpectedly, MLN4924 treatment significantly reduced the levels of VEGFR2 protein (Fig. 1A).

To clarify the effect of MLN4924 on VEGFR2 more precisely, the effects of different concentrations of MLN4924 on VEGFR2 protein and mRNA levels in HUVECs were examined. After the HUVECs were cultured with 0.1, 0.3 or 0.6 μM MLN4924 for 72 h, Western blotting and qRT-PCR analyses were performed. The results showed that both the levels of VEGFR2 protein and mRNA decreased in a dose-dependent manner (Fig. 1B). This suppression largely occurred at concentrations >0.3 μM MLN4924 (up to 80% inhibition) (Fig. 1C).

VEGF-A induces the expression of several angiogenesis-regulating molecules through VEGFR2, such as PTGS2 (cyclooxygenase-2) and vascular cell adhesion molecule-1 (VCAM1)^18^. To estimate the effect of VEGF stimulation on MLN4924-treated HUVECs, qRT-PCR assessments for *PTGS2* and *VCAM1* were performed. After the HUVECs were cultured with 0.3 μM MLN4924 or DMSO for 72 h, the cells were stimulated with VEGF-A for 2 h. *PTGS2* and *VCAM1* mRNA levels were elevated 5.7- and 3.2-fold by VEGF-A stimulation, respectively (Fig. 1D). However, these inductions were almost completely abrogated to basal levels by MLN4924 treatment (Fig. 1D). These results indicated that the inhibition of VEGF-A-induced activation of endothelial cells by MLN4924 treatment was due to the depletion of *VEGFR2* mRNA and the resulting downregulation of VEGFR2 protein.

### CUL3 was involved in *VEGFR2* mRNA expression and VEGF signaling

**Figure 2:**
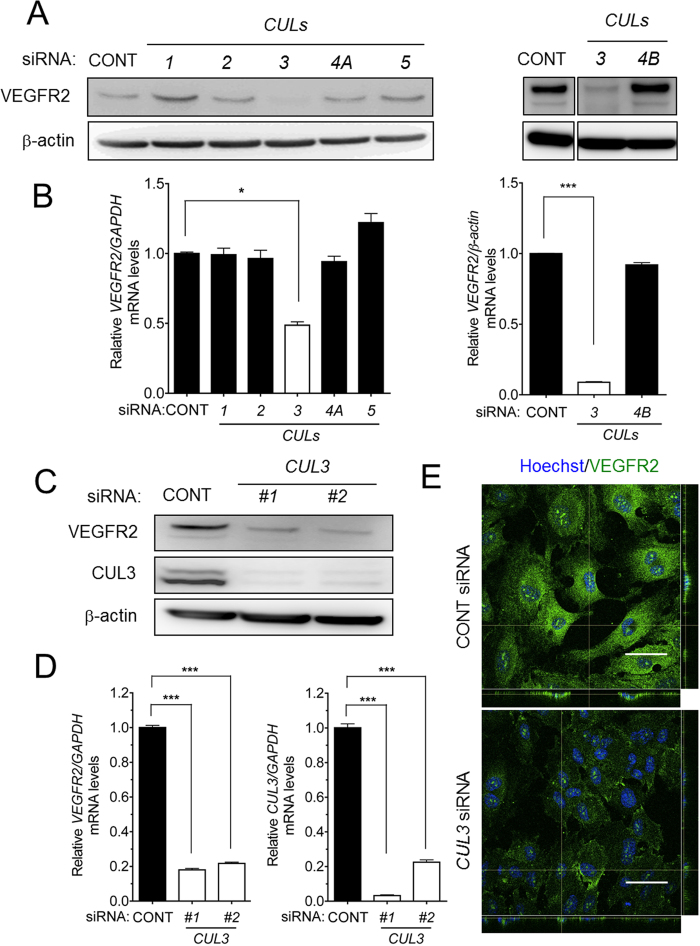
CUL3 regulated *VEGFR2 mRNA* expression in HUVECs. (**A**) HUVECs were transfected with Mission siRNAs (Sigma-Aldrich) or ON-TARGET plus Smart pool siRNA (Dharmacon) against CONT*, CUL1, CUL2, CUL3, CUL4A* and *CUL5*, or *CONT, CUL3* and *CUL4B*, and then cultured for 72 h. The proteins were extracted and separated using 10% SDS-polyacrylamide gels. VEGFR2 proteins were then detected by Western blotting using β-actin as a loading control. (**B**) *VEGFR2* mRNA levels were quantified by qRT-PCR in CONT*, CUL1, CUL2, CUL3, CUL4A* or *CUL5* (Sigma-Aldrich)-, and CONT*, CUL3* or *CUL4B* siRNAs (Dharmacon)-transfected HUVECs, and normalized to *GAPDH* or β-actin mRNA levels. *p < 0.05. (**C**) HUVECs were transiently transfected with control siRNA (*left lane*), *CUL3* siRNA#1 (*middle lane*), and *CUL3* siRNA#2 (*right lane*) (Sigma-Aldrich). The cells were harvested 72 h after transfection, and the proteins were analyzed by Western blotting using anti-VEGFR2 and anti-CUL3 antibodies. Anti-β-actin was used as an internal control. (**D**) *CUL3* siRNA#1 or #2 were transfected into HUVECs and cultured for 72 h. *VEGFR2 (left panel*) and *CUL3* mRNA (*right panel*) levels were measured by qRT-PCR. ***p < 0.001. (**E**) HUVECs cultured on gelatin-coated coverslips were transfected with control or *CUL3* siRNA. The cells were fixed and stained with anti-VEGFR2 antibody, followed by Alexa 488-conjugated goat anti-rabbit IgG. Scale bar: 50 μM. The experiments were performed independently at least 3 times.

CUL UbE3 ligases require modification by NEDD8 for their activation^6^, and the effect of MLN4924 on *VEGFR2* expression and its signaling in HUVECs appears to be caused by CUL inactivation. Therefore, we investigated the involvement of six CULs (CUL1, 2, 3, 4A, 4B and 5) in MLN4924-induced abrogation of *VEGFR2* expression; the effects of *CUL7* and *PARC* were not assessed because their expression levels are very low in HUVECs according to values reported in a public database (http//:157.82.78.238/refexa/main search.jsp). Mission siRNAs (Sigma-Aldrich) targeting *CUL1, 2, 3, 4A* or *5*, and ON-TARGET plus Smart pool *CUL3* or *4B* siRNA (Dharmacon) were applied to HUVECs prior to the detection of VEGFR2 protein and mRNA by Western blotting and qRT-PCR, respectively. When the HUVECs were transfected with 20 nM of each *CUL* siRNA, the production of each CUL mRNA was significantly decreased based on semi-quantitative RT-PCR and qRT-PCR analyses (Fig. S1). Interestingly, *CUL3* siRNA alone, but not other CUL siRNAs, significantly suppressed VEGFR2 protein production (Fig. 2A) and mRNA expression (Fig. 2B). *VEGFR2* expression levels following siRNA knockdown of *CUL1, 2, 3,4A* or *5* (Sigma-Aldrich) were normalized to *GAPDH* levels, whereas *VEGFR2* expression levels following siRNA knockdown of *CUL3* or *4B* (Dharmacon) were normalized to β-actin levels because *CUL4B* knockdown affected *GAPDH* mRNA levels. We further confirmed the specificity of *CUL3* knockdown on the reduction of VEGFR2 protein (approximately 80%) and mRNA (approximately 70%) levels by using two types of siRNAs for *CUL3* (Fig. 2C and D). During *CUL3* knockdown, we also performed immunofluorescence staining with an antibody against VEGFR2. As shown in Fig. 2E, compared with control cells, *CUL3* knockdown resulted in a marked decrease in the immunofluorescence intensity of VEGFR2 on the *CUL3*-depleted endothelial cell surface.

**Figure 3:**
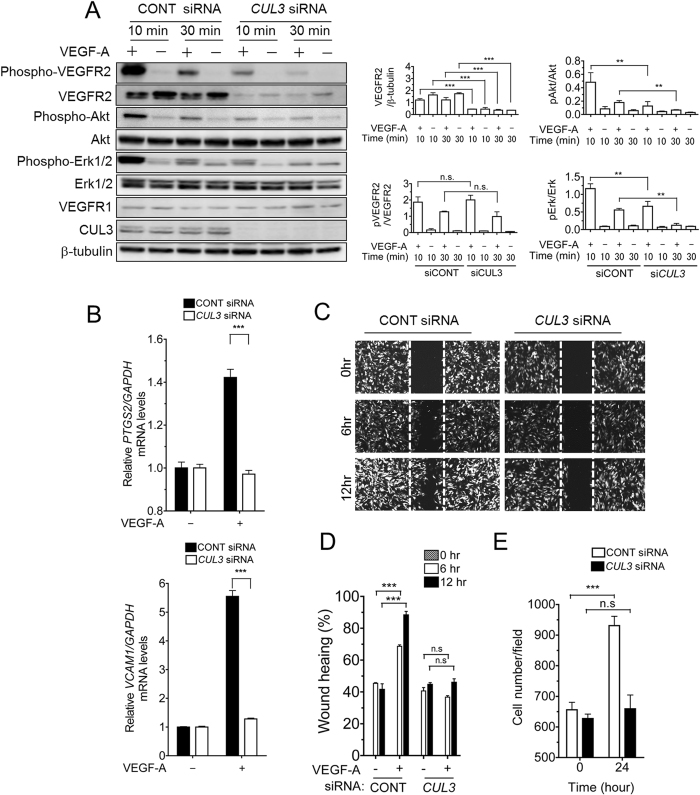
Depletion of *CUL3* abrogated VEGF-A-induced endothelial cell functions. (**A**) Control or *CUL3* siRNA-transfected HUVECs were incubated for 72 h. After serum starvation for 12 h, the cells were harvested after stimulation by VEGF-A (50 ng/mL) for 10 or 30 min. The levels of phosphorylated (p-VEGFR2, p-Erk1/2 and p-Akt) and total VEGFR1, VEGFR2, Erk, Akt and CUL3 were determined by Western blot analysis. β-tubulin was used as an internal control. The protein levels of VEGFR2, and the phosphorylated levels of VEGFR2, Akt and Erk, were quantified using ImageJ software (*right panels*). **p < 0.01. (**B**) After incubating the control or *CUL3 siRNA*-treated HUVECs for 72 h, the cells were stimulated with VEGF-A (50 ng/mL) for 2 h. The mRNA levels of *PTGS2 (upper panel*) and *VCAM1 (lower panel*) were measured by qRT-PCR. The data were normalized to the mRNA levels of *GAPDH*. ***p < 0.001. (**C**) Control or *CUL3* siRNA-transfected and Azami-Green-expressing HUVEC monolayers were wounded with a 200-μL pipette tip. After VEGF-A (50 ng/mL) stimulation for 6 or 12 h, fluorescence images were captured using a microscope. (**D**) Quantification of the motility of *CUL3* knockdown cells was conducted by measuring the distance migrated compared with the controls. ***, p < 0.001. (**E**) Control or *CUL3* siRNA-transfected HUVECs were stimulated with VEGF-A (50 ng/ml) for 24 h. After staining with Hoechst, the number of nuclei was counted. ***p < 0.001. The experiments were performed independently at least 3 times.

To elucidate the role of the *CUL3* gene in the VEGFR signaling cascade, we examined the signal transduction pathway of VEGFRs using *CUL3*-depleted HUVECs. Akt and Erk1/2 protein levels were not affected by *CUL3* knockdown, whereas VEGFR-2 levels were significantly decreased, and VEGFR-2 phosphorylation was rarely detected following VEGF-A treatment for 10 or 30 min (Fig. 3A). Similar results were obtained for MLN4924-treated HUVECs. Hence, activation of Akt and Erk1/2 downstream of VEGFR2 was completely inhibited by *CUL3* siRNA (Fig. 3A). Interestingly, VEGFR1, which is known as a decoy receptor of VEGFR2^19^, was not affected by *CUL3* knockdown (Fig. 3A).

We also investigated whether VEGF-VEGFR2 signaling in *CUL3*-depleted HUVECs affected VEGF-A-induced pro-angiogenic gene expression. As demonstrated by the qRT-PCR analyses shown in Fig. 3B, induction of the pro-angiogenic genes *PTGS2* and *VCAM1* by VEGF-A stimulation for 2 h was completely abrogated in *CUL3*-depleted HUVECs. Furthermore, to evaluate the role of *CUL3* in VEGF-mediated endothelial cell function, wound healing and cell proliferation assays were performed. Control or *CUL3* siRNA-transfected HUVECs were cultured in the presence or absence of human recombinant VEGF-A (50 ng/mL). As shown in Fig. 3C–E, VEGF-A-induced endothelial cell migration and proliferation were almost completely inhibited by *CUL3* depletion compared with their controls. Taken together, our results suggested that NEDD8-conjugated CUL3 was essential for the expression of *VEGFR2*, but not *VEGFR1*, and VEGF-A-induced angiogenic responses.

These findings indicated that among the 8 members of the CUL family, CUL3 plays a selective and crucial role in *VEGFR2* expression and VEGF signaling in HUVECs.

### SPOP, an adaptor protein for CUL3, was required for VEGFR2 expression

CUL3 assembles with Roc1/Rbx1/Hrt1 at the C-terminal region and with a Bric-a-brac/Tramtrack/Broad complex (BTB) domain-containing protein (BTBP) at the N-terminal region to form UbE3 ligase complexes. In these complexes, BTBPs play a crucial role in determining the substrate specificity^20^. One hundred eighty-three genes have been reported to encode BTBPs in human^21^. These findings suggest that one of the CUL3-BTBP complexes could target one or more VEGFR2 expression-downregulating factors for ubiquitination and subsequent degradation.

**Figure 4:**
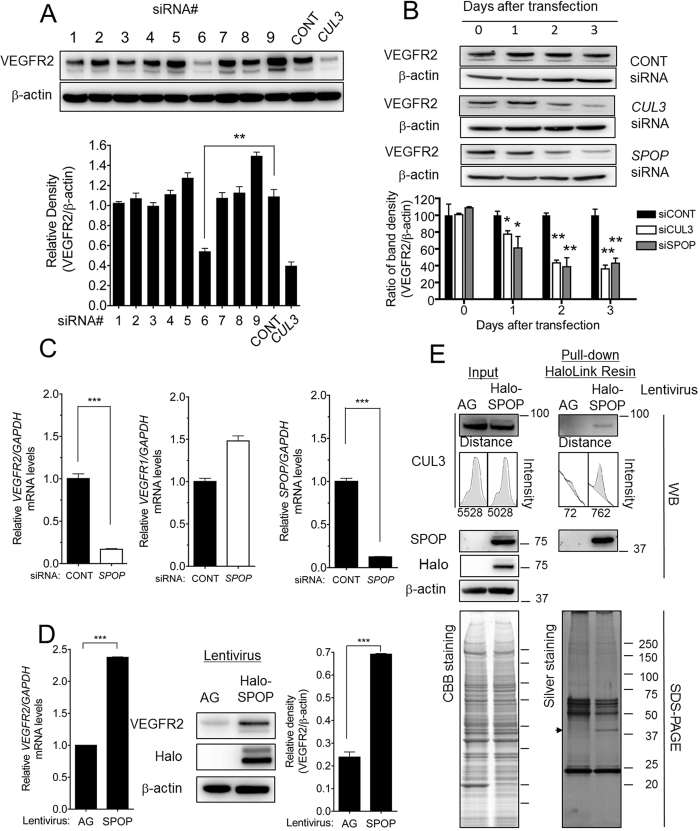
SPOP, one of the BTBPs that associates with CUL3, regulated VEGFR2 expression. (**A**) HUVECs were transfected with nine selected *BTBP* siRNAs and cultured for 72 h. After harvesting the HUVECs, the proteins were subjected to SDS-PAGE followed by Western blotting using antibodies against VEGFR2 and β-actin. The *upper panel* shows the Western blotting images of VEGFR2 and β-actin proteins in the BTBP knockdown HUVECs. The *lower panel* shows the relative band density values, which were quantified statistically using ImageJ software. Control or *CUL3* siRNAs were used as negative and positive controls, respectively. (**B**) Control, *CUL3* or *SPOP* siRNA-transfected HUVECs were collected after incubation for 0, 1, 2 or 3 days. VEGFR2 was detected by Western blotting. The relative intensity of each band was analyzed statistically using ImageJ software and normalized to β-actin (loading control). (**C**)*VEGFR2 (left panel*), *VEGFR1 (middle panel*) and *SPOP (right panel*) mRNA levels in control or *SPOP* siRNA-transfected HUVECs were quantified by qRT-PCR. The target mRNAs levels were normalized to *GAPDH* mRNA. ***, p < 0.001. (**D**) HUVECs were infected with Azami-Green- (AG) or Halo-tagged SPOP-expressing lentiviruses at a multiplicity of infection (m.o.i.) of 3. The HUVECs were harvested at 72 h after infection. VEGFR2 mRNA and protein were detected using qRT-PCR (*left panel*) and Western blot analysis (*middle and right panel*), respectively. ***p < 0.001. (**E**) Azami-Green- or Halo-tagged SPOP-expressing HUVECs were treated with NP-40 cell lysis buffer, followed by incubation with Halo-link Resin. SPOP and SPOP-associated proteins were eluted with TEV protease. Total protein and purified proteins were subjected to SDS-PAGE, and Western blotting was performed with anti-CUL3 and SPOP antibodies (*upper panel*). Input and pull-down materials were visualized by CBB (*lower left panel*) and silver staining (*lower right panel*), respectively. The arrowhead indicates SPOP released from HaloLink resin beads. The band intensities were plotted, and the peak areas, shown in gray color, were quantified using ImageJ software. The experiments were performed independently at least 3 times.

To identify the BTBP(s) responsible for acting as an adaptor for CUL3 and regulating *VEGFR2* expression, we selected 9 highly expressed BTBP genes in HUVECs using a public database (http//:157.82.78.238/refexa/main search.jsp) and performed mRNA knockdown experiments with siRNAs at a concentration of 20 nM. The Western blotting results for VEGFR2 protein in HUVECs (Fig. 4A and Table 1) revealed a decreased signal intensity of VEGFR2 in speckle-type POZ protein (SPOP)-depleted HUVECs. Monitoring of VEGFR2 protein levels by Western blotting revealed a gradual decrease to a level equal to that observed in cells treated with *CUL3* siRNA for 72 h (approximately 70–80%) (Fig. 4B). *SPOP* siRNA reduced the *SPOP* mRNA levels by more than 80%. Under this condition, *SPOP* siRNA also reduced *VEGFR2* mRNA levels by approximately 80%, whereas the levels of *VEGFR1* mRNA exhibited a relative increased (Fig. 4C). By contrast, in HUVECs overexpressing SPOP (via lentiviral infection), there was a significant increase in the levels of *VEGFR2* mRNA (approximately 2- to 2.5-fold) and protein (approximately 3- to 4-fold) (Fig. 4D). Moreover, the interaction between CUL3 and SPOP in HUVECs was analyzed by pull-down assays using Halo-tag technology. Endogenous CUL3 was detected in proteins purified by Halo-tagged SPOP-conjugated resin using Western blotting (Fig. 4E). The 42-kDa band corresponding to SPOP was released from the Halo-tag resin complex by TEV protease, and it was clearly detected in the lane containing SPOP-expressing HUVECs by silver staining and Western blotting with an anti-SPOP antibody. The other specific band was not observed in the control lane. These data suggested that CUL3 associated with SPOP, which positively regulated *VEGFR2* expression in HUVECs.

### **DAXX was a substrate for CUL3-SPOP and regulated** ***VEGFR2*** **expression**

**Figure 5:**
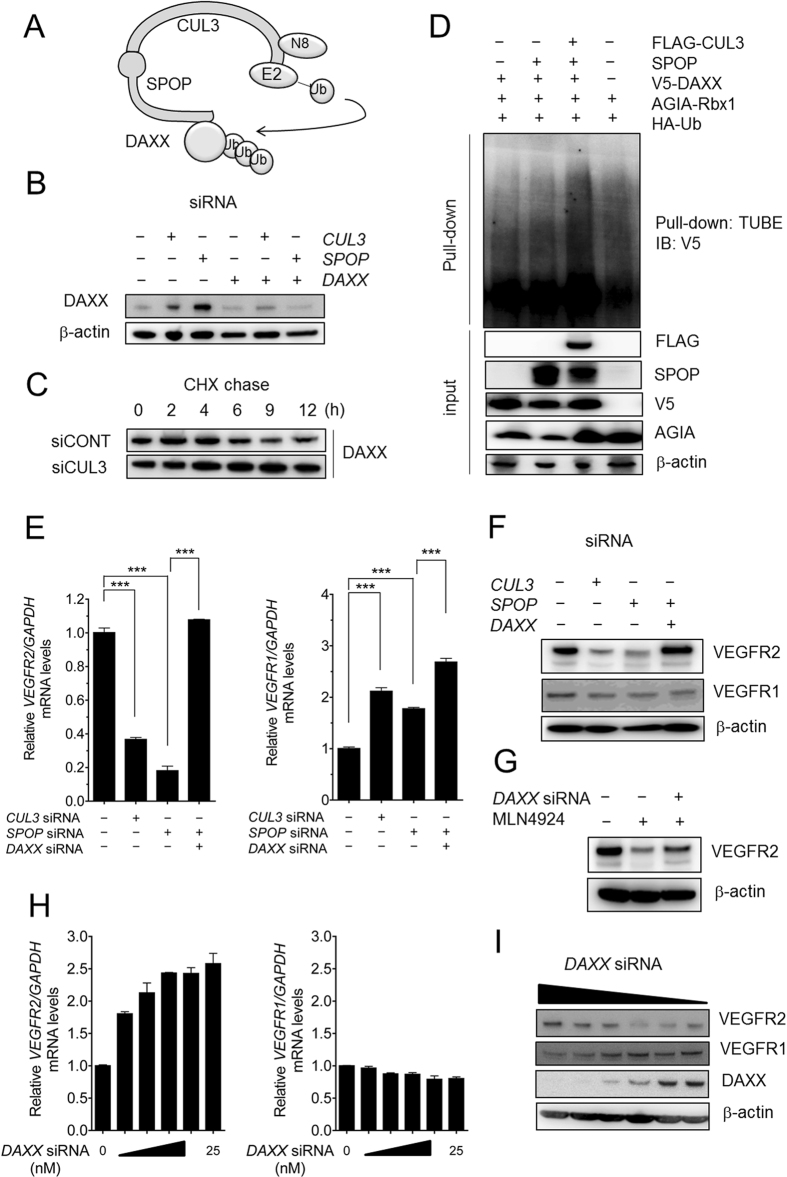
DAXX, a substrate of the CUL3-SPOP complex, negatively regulated *VEGFR2* expression. (**A**) Simplified diagram illustrating the components of the CUL3-SPOP-DAXX axis. (**B**) HUVECs were transfected with 20 nM *CUL3* or *SPOP* siRNA. HUVECs were also transfected with 20 nM of *DAXX* siRNA alone or together with 20 nM of *CUL3* or *SPOP* siRNA. The cell extracts were collected on the third day after transfection followed by Western blotting with an antibody against DAXX or β-actin. (**C**) HUVECs were transfected with CONT siRNA or CUL3 siRNA and cultured for 72 h, followed by cycloheximide treatment for 0, 2, 4, 6, 9, and 12 h. After harvesting the HUVECs, the proteins were subjected to SDS-PAGE followed by western blotting with antibodies against DAXX and β-actin. (**D**) The 293T cells were transfected with 2 μg of several combinations of constructs, as described in (**D**). The poly-ubiquitinated proteins were purified by TUBE and analyzed with anti-V5-tagged antibody. (**E**) HUVECs were transfected with 20 nM *CUL3* or *SPOP* siRNA. HUVECs were also transfected with 20 nM of *DAXX* siRNA alone or together with 20 nM of *CUL3* or *SPOP* siRNA. The amounts of *VEGFR2 (left panel*) and *VEGFR1* mRNA (*right panel*) were determined by qRT-PCR. ***p < 0.001. (**F**) HUVECs were treated with 20 nM *DAXX* siRNA alone or together with 20 nM *CUL3* or *SPOP,* proteins were extracted and VEGFR2 and VEGFR1 were detected by Western blotting. β-actin was used as an internal control. (**G**) After transfection with control or *DAXX* siRNA, HUVECs were cultured with DMSO or MLN4924 (0.3 μM) for 72 h. HUVECs were harvested, and VEGFR2 and β-actin proteins were detected by Western blotting. (**H**) HUVECs were treated with *DAXX* siRNA at various concentrations (0, 1.56, 3.13, 6.25, 12.5 or 25 nM). *VEGFR2 (left panel*) and *VEGFR1 (right panel*) mRNA levels were quantified by RT-PCR. (**I**) Proteins extracted from *DAXX* siRNA (0, 1.56, 3.13, 6.25, 12.5 or 25 nM)-transfected HUVECs were subjected to SDS-PAGE, followed by Western blotting using anti-VEGFR1, anti-VEGFR2, anti-β-actin and anti-DAXX antibodies.

*VEGFR2* mRNA expression in HUVECs was suppressed by MLN4924, *CUL3* siRNA and *SPOP* siRNA. These results suggested that the neddylated-CUL3-SPOP complex indirectly regulated *VEGFR2* mRNA expression. The molecular mechanism underlying this regulation, however, is still unclear. Therefore, we speculated that a direct substrate of CUL3-SPOP UbE3 ligase would be able to regulate *VEGFR2* mRNA expression. Several proteins have been reported to be substrates of CUL3-SPOP UbE3 ligase^20^. Among them, we analyzed DAXX protein levels under the conditions described in Fig. 5A. The Western blotting results (Fig. 5B) showed that the levels of endogenous DAXX protein increased following *SPOP* or *CUL3* knockdown. This upregulation was completely abrogated by *DAXX* knockdown (Fig. 5B). In addition, a cycloheximide chase assay revealed that the stability of DAXX protein was dramatically increased by CUL3 depletion (Fig. 5C), and an *in vivo* ubiquitination assay revealed that CUL3 and SPOP overexpression significantly increased the poly-ubiquitination of DAXX (Fig. 5D). If DAXX indeed plays a crucial role in *VEGFR2* mRNA expression, we hypothesized that the knockdown of *DAXX* would rescue the depletion of *VEGFR2* mRNA by *SPOP* siRNA. To test this hypothesis, rescue experiments were performed. Transfection of *SPOP* siRNA together with *DAXX* siRNA resulted in the complete recovery of both the mRNA and protein levels of VEGFR2 (Fig. 5E and F). However, the levels of VEGFR1 protein in *SPOP* knockdown HUVECs were not affected by co-transfection with *DAXX* siRNA, although their mRNA levels were slightly upregulated (approximately 1.5-fold) (Fig. 5E and F). In addition, the VEGFR2 protein levels in MLN4924-treated HUVECs were also partially recovered by *DAXX* knockdown (Fig. 5G). Moreover, to confirm whether DAXX could be a master regulator of *VEGFR2* expression, we measured the levels of VEGFR2 in various concentrations of *DAXX* siRNA-transfected HUVECs. qRT-PCR showed that the *VEGFR2* mRNA level was dramatically increased by *DAXX* siRNA treatment in a dose-dependent manner, and a 25 nM dose resulted in increased levels of VEGFR2 (2.5-fold higher than in the absence of *DAXX* siRNA; Fig. 5H). In contrast to the *VEGFR2* level, the *VEGFR1* mRNA level was slightly decreased in a dose-dependent manner. The corresponding protein levels of VEGFR2, VEGFR1 and DAXX were determined by Western blot analysis (Fig. 5I). These results suggest that CUL3-SPOP regulated VEGFR2-mediated endothelial cell functions by controlling the level of DAXX. However, the pathway linking DAXX to *VEGFR2* mRNA remains unknown.

### **The CUL3-SPOP-DAXX axis regulated the expression of genes essential for angiogenesis**

**Figure 6:**
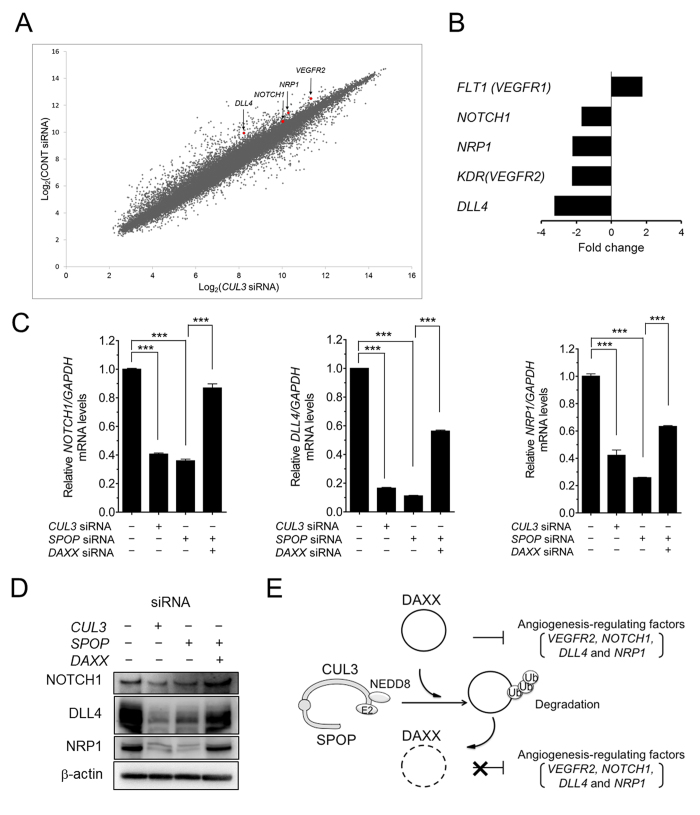
The CUL3-SPOP-DAXX axis positively regulated *NOTCH1, NRP1* and *DLL4* expression. (**A**) DNA microarray analyses (log_2_ signal intensity) of control siRNA compared with *CUL3* siRNA. (**B**) Five genes were selected from the microarray data. The relative microarray expression levels are shown; fold-change ratios for the microarray analysis were calculated considering the expression levels of *GAPDH*. (**C**) HUVECs were cultured with 20 nM *CUL3* or *SPOP* siRNA alone. HUVECs were also cultured with 20 nM *SPOP* together with *DAXX* siRNA. *NOTCH1 (left panel*), *DLL4 (middle panel*) and *NRP1 (right panel*) mRNA levels were quantified by RT-PCR. ***p < 0.001. (**D**) HUVECs were treated with 20 nM control, *CUL3* or *SPOP* siRNA alone or *SPOP* together with *DAXX* siRNA. Proteins were extracted from the transfected HUVECs, and NOTCH1, DLL4 and NRP1 were detected by Western blotting. (**E**) Schematic of the molecular mechanism. DAXX suppresses pro-angiogenic factors, including VEGFR2, NOTCH1, DLL4 and NRP1. When DAXX is degraded by the CUL3-SPOP complex, the expression of pro-angiogenic genes is upregulated, resulting in the progression of angiogenesis.

DAXX has been shown to be involved in the regulation of the cell cycle and apoptosis via the expression of various genes^22^, suggesting that a wide range of target genes are regulated by the CUL3-SPOP-DAXX axis in HUVECs. To investigate in greater detail whether the CUL3-SPOP-DAXX axis could target other genes, we first performed comparative gene expression profiling of control and *CUL3* siRNA-treated HUVECs using a DNA microarray. As shown in Fig. 6A, many genes were up- and downregulated in response to *CUL3* knockdown. Among the genes that were downregulated more than two-fold, we discovered three of particular interest: *NOTCH1, DLL4* and neuropilin1 (*NRP1*), which are important in angiogenic signaling. DLL4 is a Notch ligand, and DLL4-Notch1 signals negatively regulate angiogenesis, whereas NRP1 is a binding protein of VEGFs and functions as a co-receptor of VEGFR2. The mRNA levels of *NOTCH1, DLL4* and *NRP1* were validated by qRT-PCR and estimated to be reduced by approximately 60% in the control in *CUL3*-depleted HUVECs (Fig. 6A,B). We further analyzed whether the expression of these genes was also regulated by the CUL3-SPOP-DAXX axis. qRT-PCR analyses were performed, and the directional fold-change of each gene was confirmed. Significant decreases in *NOTCH1, DLL4* and *NRP1* were observed in both *CUL3* and *SPOP* knockdown HUVECs (Fig. 6C). In addition, we observed that the suppression of each gene by *SPOP* knockdown was largely reversed by transfection of *DAXX* siRNA (Fig. 6C). As shown in Fig. 6D, the corresponding changes in proteins were confirmed. Taken together, these findings strongly suggest that the CUL3-SPOP-DAXX axis regulates angiogenesis by upregulating not only VEGF-VEGFR2-NRP1 but also DLL4-Notch 1 signaling (Fig. 6E).

In the present study, the molecular mechanisms by which the CUL3-SPOP-DAXX axis positively regulates *VEGFR*2 mRNA remain unclear. Therefore, to assess the role of the neddylated-CUL3-SPOP-DAXX axis on *VEGFR2* mRNA metabolism, luciferase promoter assays and actinomycin D chase assays were performed because both transcriptional and post-transcriptional regulations are essential for *VEGFR2* gene expression. As shown in Fig. S2, however, there were no significant decreases in the *VEGFR2* promoter activity when HUVECs were treated with *CUL3* siRNA or *SPOP* siRNA (Fig. S2). In addition, the stability of *VEGFR2* mRNA was only slightly decreased by *CUL3* and *SPOP* knockdown (Fig. S3). Similar results were obtained for *NRP1, NOTCH1* and *DLL4* mRNAs (Fig S3). Based on these findings, it can be speculated that the neddylated-CUL3-SPOP-DAXX axis may regulate pro-angiogenic genes such as VEGFR2 via epigenetic regulation. To test this hypothesis, we harvested *CUL3* knockdown, *SPOP* knockdown and *SPOP* knockdown together with *DAXX* knockdown HUVECs. We investigated major histone modifications (methylation of histone H3 at lysine 4 (H3K4me3), methylation of histone H3 at lysine 27 (H3K27me3) and acetylation of histone H3 at lysine 9 (H3K9Ac)) in the promoter regions of target genes by chromatin immunoprecipitation assays. Herein, we provide the first evidence that the chromatin status of these promoters was monovalent (H3K4me3) because of the weak enrichment for H3K27me3 similar to the negative controls, compared with H3K4me3 and H3K9Ac (data not shown). Among the histone modifications, we found that trimethylated forms of histone H3 at lysine 4 (H3K4me3; associated with active chromatin and gene expression) were dramatically reduced by *CUL3* and *SPOP* knockdown in the *VEGFR2, DLL4, Nrp1,* and *Notch1*, but not *VEGFR1*, genes (Fig. S4). These histone modification changes were correlated with their mRNA expression levels (Figs 5E and 6C). However, knockdown of additional *DAXX* did not rescue the depletion of trimethylated forms in target genes by *SPOP* siRNA. These results suggest that the CUL3-SPOP axis targets a critical regulator of angiogenic gene expression via histone modification at H3K4me3, and the epigenetic regulator and DAXX may regulate, in a complementary manner, the mRNA levels of angiogenic factors such as *VEGFR2, DLL4, Nrp1,* and *Notch1*.

## Discussion

The VEGFR2 signaling pathway is important for both vasculogenesis and angiogenesis. Genetic deletion of *VEGFR2* from mice results in a complete absence of blood vessels, resulting in embryonic lethality in haploinsufficient animals^23^,^24^. Moreover, a large number of studies have demonstrated that pharmacological blockage of VEGF-VEGFR2 signaling can suppress tumor angiogenesis, resulting in decreased tumor growth *in vivo*. However, a significant number of such patients do not respond to anti-VEGF therapy when used either as a single agent or in combination with chemotherapy; the benefit is relatively short-lived, and the majority of patients relapse and progress^25^. The vasculature of tumors is abnormal and dysfunctional, displaying dilatation, tortuousness and leakiness, which are characteristics of an imbalance between pro- and anti-angiogenic signals^26^.

Consequently, it is essential to elucidate the regulatory mechanisms underlying the expression and activation of VEGFR2 and to establish more promising anti-angiogenic therapies. In the present study, we provide the first evidence that a neddylation inhibitor (MLN4924) blocks VEGF-A-induced activation of kinases and gene expression by inactivating CUL3-mediated *VEGFR2* expression. These findings are completely correlated with the anti-angiogenic phenotypes related to MLN4924 treatment^7^ or CUL3 knockdown^16^. However, we found a discrepancy between MLN4924- and *CUL3* siRNA-treated HUVECs in terms of VEGF-A-induced Akt phosphorylation. The basal levels of Akt phosphorylation were increased in MLN-4924-treated HUVECs. A recent study has revealed a large number of NEDD8-target molecules related to cell survival using a proteomic approach in A375 melanoma cells^27^. Furthermore, phosphorylated levels of Akt were increased in response to MLN4924 treatment in MCF7 and HeLa cell^28^. By contrast, in HCT116 cells, phosphorylation was decreased. These reports and our data suggest that MLN4924 may modulate Akt phosphorylation levels via an unknown cell type-specific molecular mechanism, excluding CUL3-mediated signaling.

It is known that CUL3 associates with BTBPs that determine substrate specificity. Several CUL3-BTBP-based E3 ligase complexes have been reported to be involved in various events. For example, CUL3-Keap1-Nrf2 is essential for oxidant regulation^29^, CUL3-BAZF-CBF1 is important for angiogenic switching^16^ and CUL3-SPOP-Gli1/Gli2 participates in morphological determination during developmen^30^,^31^,^32^. Among the BTBPs, SPOP has been relatively well characterized as a key adaptor protein for CUL3-based ubiquitin ligases. In the present study, we found that knockdown of *SPOP* down-regulated *VEGFR2* mRNA and protein levels (Fig. 4) and that endogenous CUL3 interacted with SPOP in HUVECs. These experimental outcomes of *SPOP* knockdown were blocked by knocking down *DAXX.* However, VEGFR2 down-regulation by MLN4924 was not completely rescued by DAXX knockdown. Recent evidence has shown that gene-interacting proteins, such as MLX, EID1, KLF5, ORC6L, MAGEA6, MORF4L2, MRFAP1, MORF4L1, and TAX1BP1, are NEDD8 target protein^27^, indicating that MLN4924 broadly modulates transcriptional signaling, targeting CUL3-independent post-transcriptional pathways. Therefore, the identification of other target molecule(s) of NEDD8 with regulatory activity towards the angiogenic expression of genes, such as VEGFR2 will be need as a future study. These results suggest that among the downstream NEDD8 signaling pathways, CUL3-SPOP-DAXX is a novel regulatory axis in VEGFR2 gene expression. We also showed that the CUL3-SPOP-DAXX axis was necessary for *NRP1, DLL4* and *NOTCH1*, but not *VEGFR1*, gene expression.

NRP1 binds to VEGF-A and acts as a co-receptor of VEGFRs^33^. The signal outputs of the VEGF-A-VEGFR2 pathway are enhanced by the interaction with NRP1. However, DLL4-Notch 1 signaling is also critical for vascular sprouting and stabilization^34^,^35^. During angiogenesis, DLL4 in tip cells activates Notch signaling in stalk cells, suppressing the expression of tip cell-specific genes such as *DLL4* and *VEGFR2*. A recent investigation showed that during retinal angiogenesis, inhibition of Notch signaling by a specific inhibitor of γ-secretase DAPT or genetic deletion of *DLL4*, led to hyper-vascularization due to upregulation of VEGF-VEGFR2 signaling^36^. These results indicated that the collaboration between VEGF and Notch signaling is essential for angiogenic sprouting. The data presented herein showed that the CUL3-SPOP-DAXX axis could function as a crucial angiogenic mediator through the regulated expression of *VEGFR2, NRP1, NOTCH1* and *DLL4*.

However, the CUL3-SPOP-DAXX axis had no effect on *VEGFR1* expression. VEGFR1 is highly expressed on endothelial cells and associates with VEGF-A, VEGF-B, and placenta growth facto^37^. A *VEGFR1* deficiency results in early embryonic lethality characterized by vascular abnormalities with overgrowth phenotypes of endothelial cells^23^,^38^. Moreover, the binding affinity of VEGFR1 for VEGF-A is higher than that of VEGFR2, but its tyrosine kinase activity is weaker than that of VEGFR2. Therefore, VEGFR1 has been proposed to negatively modulate angiogenesis by acting as a decoy receptor of VEGFR2.

These findings suggest that the regulation of pro-angiogenic VEGF-VEGFR2-NRP1 and anti-angiogenic DLL4-Notch1 can be coupled, but VEGF-VEGFR2-NRP1 and VEGF-VEGFR1 signaling are functionally distinct and differentially regulated. Based on our data, we propose that the CUL3-SPOP-DAXX axis can positively and effectively regulate angiogenesis by upregulating and balancing VEGF-VEGFR2-NRP1 and DLL4-Notch 1 signaling.

An important question in this study concerned how DAXX could regulate the mRNAs of the above-described angiogenic regulators. DAXX was first identified as a Fas-binding cytosolic protein that regulates cellular apoptosis^22^. DAXX also localizes in the nucleus and acts as a transcriptional and cell cycle regulator by associating with the PML nuclear body and the nuclear heterochromatin region. DAXX carries the SPOP-binding motif ^608^ VSSTT^612^. NEDD8 modification of CUL3 is essential for the degradation of DAXX in 293T cells^39^,^40^. In the present study, the endogenous level of DAXX protein was upregulated by knockdown of *CUL3* or *SPOP* in HUVECs (Fig. 5B). We also provided evidence that DAXX negatively regulated *VEGFR2* expression. A previous study has demonstrated that DAXX can repress Ets-1, which regulates the expression of angiogenic genes, such as matrix metalloproteinases, *VEGFR1* and *VEGFR2,* by binding to unique sequences (GGAA) on their promoter^41^. Here, we showed that the CUL3-SPOP-DAXX axis positively regulated the expression of *VEGFR2* but not *VEGFR1*. In addition, luciferase promoter assays revealed that *CUL3* and *SPOP* knockdown did not affect *VEGFR2* promoter activity (Fig. S2). Based on these and the previously reported data, it seems unlikely that the CUL3-SPOP-DAXX axis regulates *VEGFR2* expression via a transcriptional factor such as Ets-1 in the −780 to + 268 of VEGFR2 promoter region. However, it is possible that the CUL3-SPOP-DAXX axis targets a region upstream of VEGFR2 genes that includes the enhancer region. Although the construct (pGL3-VEGFR2-780, Addgene) used for the luciferase promoter assay contains important cis elements, such as several Sp1 sites, two AP-2 consensus sites and an E-box, the association with DAXX occurs in a region further upstream of the VEGFR2 gene that remains to be elucidated. Further analyses using ChIP sequencing with an anti-DAXX antibody may be needed. Actinomycin D chase assays also demonstrated that the stability of *VEGFR2* mRNA was not significantly reduced by *CUL3* and *SPOP* depletion (Fig. S3). These data suggest that post-transcriptional regulation is also not required for the gene expression of *VEGFR2* by CUL3-SPOP-DAXX. On the other hand, DAXX is also known to control transcription via epigenetic regulatio^42^. Several studies have reported that DAXX directly and indirectly regulates gene expression by interacting with various chromatin remodeling proteins such as histone deacetylase, DNA methyl transferase and histone-acetyltransferase CBP/p300^43^. Furthermore, recent studies have shown that DAXX acts as an H3.3-specific histone chaperone in mammalian cells^44^. Based on these findings, we investigated the main histone modifications of the promoter regions in the *VEGFR2, VEGFR1, NOTCH1, DLL4* and *NRP1* genes. We found that the H3K4me3 levels in 4 of the 5 promoters (excluding *VEGFR1*) were dramatically decreased in response to *CUL3* or *SPOP* depletion; these changes were not rescued by additional *DAXX* knockdown (Fig. S4). Our results suggest at least two possible mechanisms of CUL3-mediated angiogenic gene induction. First, DAXX is a main *VEGFR2* mRNA regulator as a substrate of the CUL3-SPOP axis and may negatively regulate the mRNA levels of angiogenic genes via other types of epigenetic regulation such as microRNAs. Second, the CUL3-SPOP axis may target negative regulators of H3K4 trimethylation for ubiquitination and degradation. These substrates may be responsible for the complementary regulation of angiogenesis-related gene expression. Future experiments using genome-wide ChIP-Seq and microarray analyses are needed to clarify the complete molecular mechanism that regulates angiogenic gene expression. In addition, we also need showing the relevance of CUL3-SPOP-DAXX to angiogenesis through *in vivo* studies using gene-deficient mice. We believe that our data contribute to our understanding of the precise mechanism by which the CUL3-SPOP-DAXX axis regulates angiogenesis in humans and provide insights that may help to establish a new strategy for the treatment of angiogenesis-associated diseases.

## Materials and Methods

### Reagents and antibodies

Recombinant human VEGF-A was purchased from R&D Systems (Minneapolis, MN). MLN4924 was from Funakoshi Chemical Co. (Tokyo, Japan). Anti-flk1/VEGFR2 (C-1158: sc-504) and anti-SPOP (C-14: sc-66649) antibody were from Santa Cruz Biotechnology (Santa Cruz, CA). Anti-CUL3 (Clone CUL3-9, Catalog# SAB4200180), mouse monoclonal anti-β-actin antibody (clone AC-15) and anti–β-tubulin antibody (clone JDR.3B8) were from Sigma-Aldrich (St. Louis, MO). Anti–phospho-VEGFR2 (Tyr1175)(19A10; #2478), anti–phospho-Akt (Ser473; #9270), anti–Akt (#9272), anti–phospho-p44/42 MAPK (Erk1/2) (Thr202/Tyr204; #9101), anti–Erk (p44/42 MAPK (Erk1/2); #9102), anti–DAXX (25C12; #4533), anti–VEGF receptor1 (#2893) and anti–Neuropilin1 (D62C6) antibody were from Cell Signaling Technology (Danvers, MA). Anti-HaloTag monoclonal antibody (G9211) was from Promega (Madison, WI). The Wako silver staining kit was obtained from Wako Biochemicals (Osaka, Japan).

### Cell culture

Human umbilical vein endothelial cells (HUVECs) were obtained from Cell System (Kirkland, WA). HUVECs were grown in the endothelial growth medium EGM-2 (Lonza, Walkersville, MD). All experiments were performed with HUVECs at passages 2–4.

### Lentiviral vector construction

Lentiviral vectors were constructed by inserting cDNAs encoding Halo-tagged *SPOP* into the lentiviral expression vector CSII-CMV-MCS-IRES2-Bsd. Lentiviral vectors were produced in 293T cells. The expression plasmid for Halo-tagged *SPOP* (Flexi ORF Clone) was purchased from Promega. Lentiviral expression and packaging vectors were kindly provided by Dr. Miyoshi (RIKEN BioResource Center, Tsukuba, Japan).

### RNA interference

The siRNAs were obtained from Sigma-Aldrich and Dharmacon (Lafayette, CO) (listed in Supplementary Table 1). MISSION siRNA Universal Negative Control SIC-001 (Sigma-Aldrich) and siGENOME Non-Targeting siRNA (Dharmacon, D-001206-14-20) were used as control siRNAs. The siRNA transfection was performed using Lipofectamine RNAiMAX (Invitrogen, Carlsbad, CA) at 20 nM according to the manufacturer’s protocol.

### Western blotting

Twenty micrograms of protein from HUVECs were electrophoresed in sodium dodecyl sulfate-polyacrylamide gels electrophoresis (SDS-PAGE). The separated proteins were then transferred to polyvinylidene difluoride membranes that were then blocked with 5% nonfat milk in 0.05% Tween 20/PBS (−) (PBS-T) for 30 min, followed by incubation with a primary antibody (1:1000 [v/v]). After washing with PBS-T, the membrane was labeled with the appropriate horseradish peroxidase-conjugated IgG antibodies (Promega; 1:4000 [v/v]). The proteins were visualized using enhanced chemiluminescence and imaged using a LAS-4000 Image Reader (Fujifilm, Tokyo, Japan).

### Isolation of RNA, cDNA library synthesis, and quantitative RT-PCR

Total RNAs were extracted from cells using ISOGEN II (Nippon Gene, Tokyo, Japan) according to the manufacturer’s protocol. One microgram of RNA was used for cDNA synthesis using High Capacity RNA-to-cDNA Master Mix (Applied Biosystems, Foster City, CA). Real-time PCR was carried out (FastStart Universal SYBR Green Master ROX; Roche Diagnostics, Basel, Switzerland) with the ABI 7300/7500 Real-Time PCR system (Applied Biosystems). The sequences of the primers are listed in Supplementary Table 2.

### Fluorescence immunostaining

HUVECs were plated on gelatin-coated coverslips and then cultured with EGM-2 for 24 h. siRNA targeting human *CUL3* or control siRNA were transiently transfected into the HUVECs, followed by culturing for 72 h. The cells were fixed with a 4% paraformaldehyde solution for 30 min and washed three times with PBS. Triton X-100–permeabilized cells were blocked with PBS containing 4% bovine serum albumin (4% bovine serum albumin-PBS), and the cells were labeled with anti-VEGFR2 antibody (1:1000 dilution) in 4% bovine serum albumin-PBS overnight at room temperature. The cells were then washed three times with PBS and probed with Alexa Fluor 488-conjugated goat anti-rabbit IgG antibody (1:1000 dilution) (Invitrogen) in 4% bovine serum albumin-PBS for 1 h at room temperature. After washing three times with PBS, fluorescent images were obtained using a confocal laser microscope A1R (Nikon Corp, Tokyo, Japan).

### Cell migration assay

Azami-Green-expressing HUVECs were seeded onto 35-mm dishes. On the following day, control or *CUL3* siRNA was transfected into the cells and cultured for an additional 2 days. After serum starvation in endothelial cell basal medium-2 (EBM-2 containing 0.15% FBS) for 12 h, the cell culture was scratched with a 200-μL pipette tip, followed by incubation with EBM-2 containing VEGF-A or BSA for 12 h. Fluorescence images were obtained at 6-h interval using a microscope (Olympus, Tokyo, Japan).

### Cell proliferation assay

HUVECs (10^5^ cells/well) were cultured in a 6-well plate in EGM2 for 24 h. After transfection with siRNA against the control or *CUL3*, the HUVECs were incubated for 48 h, and then the medium was replaced with EBM2 containing 0.15% FCS for serum starvation. The HUVECs were cultured with 50 ng/mL of VEGF-A for 24 h, and then the nuclei were stained with Hoechst. Fluorescence images were captured using microscopy, and the signals were counted using Image Pro-Plus software (Media Cybernetics, Silver Spring, MD).

### Pull-down assay

Halo-tagged SPOP or Azami-Green-expressing HUVECs were lysed in cell extraction buffer (50 mM Tris-HCl pH7.5, 0.15 M NaCl, 0.5% NP40). The samples were sonicated three times for 30 sec. The extracts were incubated with HaloLink Resin (Promega) at 4 °C for 3 h, followed by elution of SPOP and SPOP-binding proteins using TEV protease. The pull-down material was separated by SDS-PAGE, assessed by Western blotting with anti-CUL3, anti-Halo-tag and anti-SPOP antibodies, and visualized by silver staining.

### In vivo ubiquitination assay

The 293T cells were transfected with several combinations of constructs (pcDNA3.1), such as FLAG-tagged CUL3, SPOP, V5 tagged DAXX, AGIA-tagged^45^ Rbx1 and HA-tagged ubiquitin. After culturing the cells for 48 h, MG132 (final concentration, 20 μM) was applied for 8 h to inhibit the function of the proteasome. The cells were lysed with NP40 buffer cell extraction buffer (50 mM Tris-HCl pH 7.5, 0.15 M NaCl, 0.5% NP40) and incubated with Tandem Ubiquitin Binding Entity (TUBE) (Nacalai Tesque, Kyoto, Japan). After the beads were washed with lysis buffer, the binding proteins were eluted with SDS sample buffer. Western blot analyses were performed to detect the V5-tag as described.

### Cycloheximide chase assay

HUVECs were treated with CONT or *CUL3* siRNA and then incubated for 72 h. After treatment with 25 μg/mL cycloheximide (Wako, Osaka, Japan), the cells were lysed at various time points (0, 2, 4, 6, 9, and 12 h). Western blot analyses for DAXX were performed as described.

### Microarray analysis

Total RNA was extracted from control or *CUL3* siRNA-transfected HUVECs with Isogen II. We used 500 ng of total RNA to generate double-stranded cDNA. The cDNA was transcribed with DIG-labeled nucleotides (Roche), fragmented, and hybridized to a Gene Chip Human Gene 1.0 ST Array (Affymetrix, Santa Clara, CA) according to the manufacturer’s instructions.

### Statistical analysis

Data were based on a minimum of 3 independent experiments. The results are represented as the means ± SEs. Two groups were compared using Student’s t test. Differences were considered significant if the *p*-values were less than 0.05. Statistical analyses were performed using GraphPad Prism (GraphPad Software, San Diego, CA).

## Additional Information

**How to cite this article**: Sakaue, T. *et al*. The CUL3-SPOP-DAXX axis is a novel regulator of *VEGFR2* expression in vascular endothelial cells. *Sci. Rep.* **7**, 42845; doi: 10.1038/srep42845 (2017).

**Publisher's note:** Springer Nature remains neutral with regard to jurisdictional claims in published maps and institutional affiliations.

## Supplementary Material

Supplemental Information

## Figures and Tables

**Table 1:** siRNAs used for screening BTBP that regulate VEGFR2.

BTB siRNA#	Gene symbol	Gene Name
1	*KLHL5*	Kelch-Like Family Member 5
2	*KLHL9*	Kelch-Like Family Member 9
3	*KLHL20*	Kelch-Like Family Member 20
4	*KLHL24*	Kelch-Like Family Member 24
5	*RHOBTB1*	Rho-Related BTB Domain Containing 1
6	*SPOP*	Speckle-Type POZ Protein
7	*ZBTB33*	Zinc Finger And BTB Domain Containing 33
8	*KCTD20*	Potassium Channel Tetramerization Domain Containing 20
9	*TNFAIP1*	Tumor Necrosis Factor, Alpha-Induced Protein 1 (Endothelial)

## References

[b1] Folkman J. Tumor angiogenesis: therapeutic implications. The New England journal of medicine 285, 1182–1186, doi: 10.1056/nejm197111182852108 (1971). 4938153

[b2] Carmeliet P. Angiogenesis in health and disease. Nat Med 9, 653–660, doi: Doi 10.1038/Nm0603-653 (2003). 12778163

[b3] Chamorro-Jorganes A. . MicroRNA-16 and MicroRNA-424 Regulate Cell-Autonomous Angiogenic Functions in Endothelial Cells via Targeting Vascular Endothelial Growth Factor Receptor-2 and Fibroblast Growth Factor Receptor-1. Arterioscl Throm Vas 31, 2595–U2578, doi: Doi 10.1161/Atvbaha.111.236521 (2011). PMC3226744 21885851

[b4] Agudo J. . The miR-126-VEGFR2 axis controls the innate response to pathogen-associated nucleic acids. Nature immunology 15, 54–62, doi: 10.1038/ni.2767 (2014). 24270517 PMC3896265

[b5] Shaik S. . SCF(beta-TRCP) suppresses angiogenesis and thyroid cancer cell migration by promoting ubiquitination and destruction of VEGF receptor 2. The Journal of experimental medicine 209, 1289–1307, doi: 10.1084/jem.20112446 (2012). 22711876 PMC3405505

[b6] Hori T. . Covalent modification of all members of human cullin family proteins by NEDD8. Oncogene 18, 6829–6834, doi: 10.1038/sj.onc.1203093 (1999). 10597293

[b7] Yao W. T. . Suppression of tumor angiogenesis by targeting the protein neddylation pathway. Cell death & disease 5, e1059, doi: 10.1038/cddis.2014.21 (2014). 24525735 PMC3944239

[b8] Soucy T. A. . An inhibitor of NEDD8-activating enzyme as a new approach to treat cancer. Nature 458, 732–736, doi: 10.1038/nature07884 (2009). 19360080

[b9] Soucy T. A., Smith P. G. & Rolfe M. Targeting NEDD8-activated cullin-RING ligases for the treatment of cancer. Clinical cancer research: an official journal of the American Association for Cancer Research 15, 3912–3916, doi: 10.1158/1078-0432.CCR-09-0343 (2009). 19509147

[b10] Lin J. J., Milhollen M. A., Smith P. G., Narayanan U. & Dutta A. NEDD8-targeting drug MLN4924 elicits DNA rereplication by stabilizing Cdt1 in S phase, triggering checkpoint activation, apoptosis, and senescence in cancer cells. Cancer research 70, 10310–10320, doi: 10.1158/0008-5472.CAN-10-2062 (2010). 21159650 PMC3059213

[b11] Milhollen M. A. . Inhibition of NEDD8-activating enzyme induces rereplication and apoptosis in human tumor cells consistent with deregulating CDT1 turnover. Cancer research 71, 3042–3051, doi: 10.1158/0008-5472.CAN-10-2122 (2011). 21487042

[b12] Luo Z. . The Nedd8-activating enzyme inhibitor MLN4924 induces autophagy and apoptosis to suppress liver cancer cell growth. Cancer research 72, 3360–3371, doi: 10.1158/0008-5472.CAN-12-0388 (2012). 22562464

[b13] Sarikas A., Hartmann T. & Pan Z. Q. The cullin protein family. Genome biology 12, doi: Artn 220, doi 10.1186/Gb-2011-12-4-220 (2011). 10.1186/gb-2011-12-4-220 PMC3218854 21554755

[b14] Hoeller D. & Dikic I. Targeting the ubiquitin system in cancer therapy. Nature 458, 438–444, doi: 10.1038/nature07960 (2009). 19325623

[b15] Buchwalter A. . Expression of VACM-1/cul5 mutant in endothelial cells induces MAPK phosphorylation and maspin degradation and converts cells to the angiogenic phenotype. Microvascular research 75, 155–168, doi: 10.1016/j.mvr.2007.08.004 (2008). 17950367

[b16] Ohnuki H. . BAZF, a novel component of cullin3-based E3 ligase complex, mediates VEGFR and Notch cross-signaling in angiogenesis. Blood 119, 2688–2698, doi: 10.1182/blood-2011-03-345306 (2012). 22279058

[b17] Miwa D. . Protein kinase D2 and heat shock protein 90 beta are required for BCL6-associated zinc finger protein mRNA stabilization induced by vascular endothelial growth factor-A. Angiogenesis 16, 675–688, doi: 10.1007/s10456-013-9345-x (2013). 23515950

[b18] Kim I. . Vascular endothelial growth factor expression of intercellular adhesion molecule 1 (ICAM-1), vascular cell adhesion molecule 1 (VCAM-1), and E-selectin through nuclear factor-kappa B activation in endothelial cells. The Journal of biological chemistry 276, 7614–7620, doi: 10.1074/jbc.M009705200 (2001). 11108718

[b19] Krueger J. . Flt1 acts as a negative regulator of tip cell formation and branching morphogenesis in the zebrafish embryo. Development 138, 2111–2120, doi: 10.1242/dev.063933 (2011). 21521739 PMC3082310

[b20] Lydeard J. R., Schulman B. A. & Harper J. W. Building and remodelling Cullin-RING E3 ubiquitin ligases. EMBO reports 14, 1050–1061, doi: 10.1038/embor.2013.173 (2013). 24232186 PMC3849489

[b21] Stogios P. J., Downs G. S., Jauhal J. J., Nandra S. K. & Prive G. G. Sequence and structural analysis of BTB domain proteins. Genome biology 6, R82, doi: 10.1186/gb-2005-6-10-r82 (2005). 16207353 PMC1257465

[b22] Yang X. L., KhosraviFar R., Chang H. Y. & Baltimore D. Daxx, a novel Fas-binding protein that activates JNK and apoptosis. Cell 89, 1067–1076, doi: 10.1016/S0092-8674(00)80294-9 (1997). 9215629 PMC2989411

[b23] Fong G. H., Rossant J., Gertsenstein M. & Breitman M. L. Role of the Flt-1 Receptor Tyrosine Kinase in Regulating the Assembly of Vascular Endothelium. Nature 376, 66–70, doi: 10.1038/376066a0 (1995). 7596436

[b24] Shalaby F. . Failure of Blood-Island Formation and Vasculogenesis in Flk-1-Deficient Mice. Nature 376, 62–66, doi: 10.1038/376062a0 (1995). 7596435

[b25] Rapisarda A. & Melillo G. Role of the VEGF/VEGFR Axis in Cancer Biology and Therapy. Adv Cancer Res 114, 237–267, doi: 10.1016/B978-0-12-386503-8.00006-5 (2012). 22588059

[b26] Jain R. K. . Angiogenesis in brain tumours. Nat Rev Neurosci 8, 610–622, doi: 10.1038/Nrn2175 (2007). 17643088

[b27] Liao H. . Quantitative Proteomic Analysis of Cellular Protein Modulation upon Inhibition of the NEDD8-Activating Enzyme by MLN4924. Mol Cell Proteomics 10, doi: Artn M111.009183, 10.1074/Mcp.M111.009183 (2011). PMC3226404 21873567

[b28] Zhao Y., Xiong X., Jia L. & Sun Y. Targeting Cullin-RING ligases by MLN4924 induces autophagy via modulating the HIF1-REDD1-TSC1-mTORC1-DEPTOR axis. Cell death & disease 3, e386, doi: 10.1038/cddis.2012.125 (2012). 22951983 PMC3461362

[b29] Kobayashi A. . Oxidative stress sensor Keap1 functions as an adaptor for Cul3-based E3 ligase to regulate proteasomal degradation of Nrf2. Molecular and cellular biology 24, 7130–7139, doi: 10.1128/MCB.24.16.7130-7139.2004 (2004). 15282312 PMC479737

[b30] Chen M. H. . Cilium-independent regulation of Gli protein function by Sufu in Hedgehog signaling is evolutionarily conserved. Gene Dev 23, 1910–1928, doi: Doi 10.1101/Gad.1794109 (2009). 19684112 PMC2725943

[b31] Zhang Q. . Multiple Ser/Thr-rich degrons mediate the degradation of Ci/Gli by the Cul3-HIB/SPOP E3 ubiquitin ligase. Proceedings of the National Academy of Sciences of the United States of America 106, 21191–21196, doi: 10.1073/pnas.0912008106 (2009). 19955409 PMC2795488

[b32] Wang C., Pan Y. & Wang B. Suppressor of fused and Spop regulate the stability, processing and function of Gli2 and Gli3 full-length activators but not their repressors. Development 137, 2001–2009, doi: 10.1242/dev.052126 (2010). 20463034 PMC2875842

[b33] Soker S., Takashima S., Miao H. Q., Neufeld G. & Klagsbrun M. Neuropilin-1 is expressed by endothelial and tumor cells as an isoform-specific receptor for vascular endothelial growth factor. Cell 92, 735–745, doi: Doi 10.1016/S0092-8674(00)81402-6 (1998). 9529250

[b34] Phng L. K. & Gerhardt H. Angiogenesis: a team effort coordinated by notch. Developmental cell 16, 196–208, doi: 10.1016/j.devcel.2009.01.015 (2009). 19217422

[b35] Ehling M., Adams S., Benedito R. & Adams R. H. Notch controls retinal blood vessel maturation and quiescence. Development 140, 3051–3061, doi: 10.1242/dev.093351 (2013). 23785053

[b36] Zarkada G., Heinolainen K., Makinen T., Kubota Y. & Alitalo K. VEGFR3 does not sustain retinal angiogenesis without VEGFR2. Proceedings of the National Academy of Sciences of the United States of America 112, 761–766, doi: 10.1073/pnas.1423278112 (2015). 25561555 PMC4311859

[b37] Cross M. J., Dixelius J., Matsumoto T. & Claesson-Welsh L. VEGF-receptor signal transduction. Trends in Biochemical Sciences 28, 488–494, doi: 10.1016/s0968-0004(03)00193-2 (2003). 13678960

[b38] Hiratsuka S., Minowa O., Kuno J., Noda T. & Shibuya M. Flt-1 lacking the tyrosine kinase domain is sufficient for normal development and angiogenesis in mice. Proceedings of the National Academy of Sciences of the United States of America 95, 9349–9354, doi: 10.1073/pnas.95.16.9349 (1998). 9689083 PMC21341

[b39] Kwon J. E. . BTB domain-containing speckle-type POZ protein (SPOP) serves as an adaptor of Daxx for ubiquitination by Cul3-based ubiquitin ligase. Journal of Biological Chemistry 281, 12664–12672, doi: 10.1074/jbc.M600204200 (2006). 16524876

[b40] Zhuang M. . Structures of SPOP-substrate complexes: insights into molecular architectures of BTB-Cul3 ubiquitin ligases. Molecular cell 36, 39–50, doi: 10.1016/j.molcel.2009.09.022 (2009). 19818708 PMC2847577

[b41] Murakami Y. . Ets-1-dependent expression of vascular endothelial growth factor receptors is activated by latency-associated nuclear antigen of Kaposi’s sarcoma-associated herpesvirus through interaction with Daxx. The Journal of biological chemistry 281, 28113–28121, doi: 10.1074/jbc.M602026200 (2006). 16861237

[b42] Lewis P. W., Elsaesser S. J., Noh K. M., Stadler S. C. & Allis C. D. Daxx is an H3.3-specific histone chaperone and cooperates with ATRX in replication-independent chromatin assembly at telomeres. Proceedings of the National Academy of Sciences of the United States of America 107, 14075–14080, doi: DOI 10.1073/pnas.1008850107 (2010). 20651253 PMC2922592

[b43] Salomoni P. & Khelifi A. F. Daxx: death or survival protein? Trends in cell biology 16, 97–104, doi: 10.1016/j.tcb.2005.12.002 (2006). 16406523

[b44] Elsasser S. J. . DAXX envelops a histone H3.3-H4 dimer for H3.3-specific recognition. Nature 491, 560–565, doi: 10.1038/nature11608 (2012). 23075851 PMC4056191

[b45] Yano T. . AGIA Tag System Based on a High Affinity Rabbit Monoclonal Antibody against Human Dopamine Receptor D1 for Protein Analysis. Plos One 11, doi: ARTN e0156716, 10.1371/journal.pone.0156716 (2016). PMC4894603 27271343

