# Corrigendum: The CUL3-SPOP-DAXX axis is a novel regulator of *VEGFR2* expression in vascular endothelial cells

**DOI:** 10.1038/srep46915

**Published:** 2017-12-22

**Authors:** Tomohisa Sakaue, Iori Sakakibara, Takahiro Uesugi, Ayako Fujisaki, Koh-ichi Nakashiro, Hiroyuki Hamakawa, Eiji Kubota, Takashi Joh, Yuuki Imai, Hironori Izutani, Shigeki Higashiyama

Scientific Reports 7: Article number: 42845; 10.1038/srep42845 published online: 02 20 2017; updated: 12 22 2017.

The original version of this Article contained an error in the spelling of the author Yuuki Imai, which was incorrectly given as Yu-ki Imai.

This error has now been corrected in the PDF and HTML versions of the Article, and in the accompanying Supplementary Information.

