# Supplementary material for: The CUL3-SPOP-DAXX axis is a novel regulator of VEGFR2 expression in vascular endothelial cells
Source: Sci Rep. 2017 Feb 20;7:42845. doi: 10.1038/srep42845 (PMC5317005; doi:10.1038/srep42845)
Supplement: Supplemental Information [file srep42845-s1.pdf]

Supplementary Information

The CUL3-SPOP-DAXX axis is a novel regulator of VEGFR2 expression in vascular endothelial cells

Tomohisa Sakaue, Iori Sakakibara, Ayako Fujisaki, Takahiro Uesugi, Koh-ichi Nakashiro, Hiroyuki Hamakawa, Eiji Kubota, Takashi Joh, Yu-ki Imai, Hironori Izutani and Shigeki Higashiyama

Contents

Supplementary Figure legends..................pages 2, 3

Supplementary Figures 1 to 5....................pages 4-18

Supplementary Table 1 to 3.......................pages 19-21

Supplementary Figure 1. Analysis of knockdown efficiency in *CUL*-knockdown HUVECs using RT-PCR. (A) Total RNA in C*UL1, 2, 3, 4A* or *5*-depleted HUVECs was obtained as described in Materials and Methods. RT-PCR products were separated in 1.5% agarose. The *GAPDH* gene was used as an internal control. The number of cycles of PCR was 25 and the PCR primers are listed in Supplementary Table 3. (B) qRT-PCR analyses of *CUL3* (*left panel*) and *CUL4B* mRNA *(right panel)* in *CUL3*- or *CUL4B*-knockdown HUVECs, respectively. Expression levels of target gene were normalized to β-actin levels. ***, $p < 0.001$.

Supplementary Figure 2. CUL3- or SPOP-knockdown did not affect the transcriptional activity of *VEGFR2*.

A reporter plasmid containing the firefly luciferase gene was transfected into HUVECs with pGL3-VEGFR2-780 (Addgene plasmid 21307) using X-tremeGENE HP DNA transfection reagent (Roche) according to the manufacturer's protocol. Luciferase activities were measured with a GloMax96 microplate luminometer (Promega) according to the manufacturer's instructions. The firefly luciferase activities were normalized to total cell number. *Upper panel* shows schematic representation of the luciferase reporters with the promoter of the *VEGFR2* pGL3-VEGFR2-780 plasmid. *Lower panels* show relative luciferase units (RLU) of the *VEGFR2* promoter in *CUL3*- or *SPOP*-knockdown HUVECs. The *VEGFR2* promoter reporter plasmid was transfected into HUVECs. Cells were continuously treated with CONT siRNA, *CUL3* siRNA and *SPOP* siRNA, and they were then incubated for 72 h. *VEGFR2* promoter activity was analyzed by measuring luciferase activity. The firefly luciferase activities were normalized to total cell number.

Supplementary Figure 3. Neither *CUL3*- nor *SPOP*-knockdown affected the mRNA stability of angiogenesis-regulating genes.

After HUVECs were treated with 2 mg/mL actinomycin D (Nacalai Tesque, Kyoto, Japan), the cells were lysed at various time points (0, 1, 2, 3 and 4 h). Total RNA was isolated as described above. The real-time quantitative RT-PCR was performed as described, and mRNA levels were normalized using 18S ribosomal RNA, with the mRNA level at time 0 set at 1.0. Actinomycin D chase assays for analysis of *VEGFR2, NOTCH1, NRP1* and *DLL4* mRNA stability in (A) CONT siRNA- (solid black square) and CUL3 siRNA- (red open circle) or (B) CONT siRNA- (solid black square) and SPOP siRNA- (red open circle) treated HUVECs. HUVECs were treated with

CONT, CUL3 or SPOP siRNA and then incubated for 72 h. Actinomycin D (2 μg/ml) was added immediately and the cells were incubated for the indicated times, followed by analysis of the *VEGFR2*, *NOTCH1*, *NRP1* and *DLL4* mRNA levels by qPCR. The mRNA levels were normalized to the respective 18S rRNA levels and expressed as ratio of the level at the 0-min time point.

Supplementary Figure 4. Analysis of histone modification levels at the promoter regions of angiogenesis-regulating genes in CUL3-, SPOP- or SPOP- and DAXX-knockdown HUVECs.

CUL3-, SPOP- and SPOP-knockdown together with DAXX-knockdown HUVECs were fixed with formaldehyde and the chromatin was sheared by sonication into ~200 bp fragment lengths using a Covaris System S220 (Covaris, Woburn, MA). Complexes were immunoprecipitated using antibodies specific to (A) H3K4me3 (07-473; Millipore) and (B) H3K9Ac (07-352; Millipore) or normal rabbit IgG as a negative control. DNA was purified from the isolated chromatin, and the precipitated DNA fragments were amplified by quantitative PCR. The following primers were used: human *VEGFR2*, 5′-CCTTGCTGGGCAAATAAGAG-3′ and 5′-TTGCACATCTGTGTGGTGTG-3′; human *VEGFR1*, 5′-AGCGAAGGCTGACTTAGGTG-3′ and 5′-GGACATCAGTGACCCTGACC-3′; human *GAPDH*, 5′-ACTCTGCTCTGGGTGGTCATTG-3′ and 5′-GGGTGCTGAACACTTGTAAGGAAG-3′; human *NRP1*, 5′-AAGGAGGGGAACGAGCAATG-3′ and 5′- AGCACTCGGAGGGAAAGTTG-3′; human *NOTCH1*, 5′- CTTCTTACGCAACCCCTCCC-3′ and 5′-TTGCTTCTCCGGGCCATTTA-3′; and human *DLL4*, 5′-GGGCGCAGTAACTGAATCCT-3′ and 5′- CGTGCCAAGCTTCGATGATG -3′. ns, not significant, *, $p < 0.05$, **, $p < 0.005$ and ***, $p < 0.001$.

Supplementary Figure 5. Full blots for cropped western blot data in the manuscript.

A broken line indicates approximate regions of the membranes that are included in the figures.

Supplementary Figure 1. (Higashiyama)

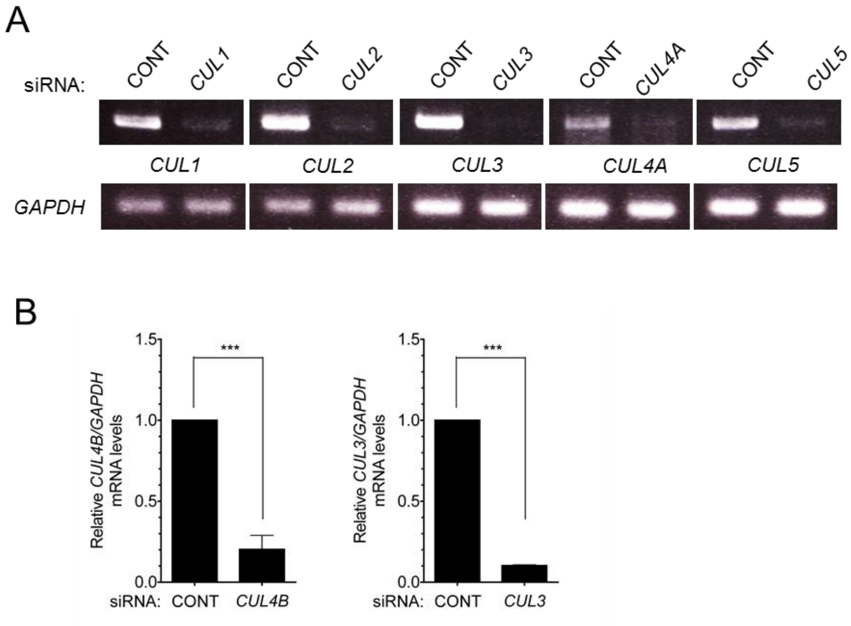

Supplementary Figure 2. (Higashiyama)

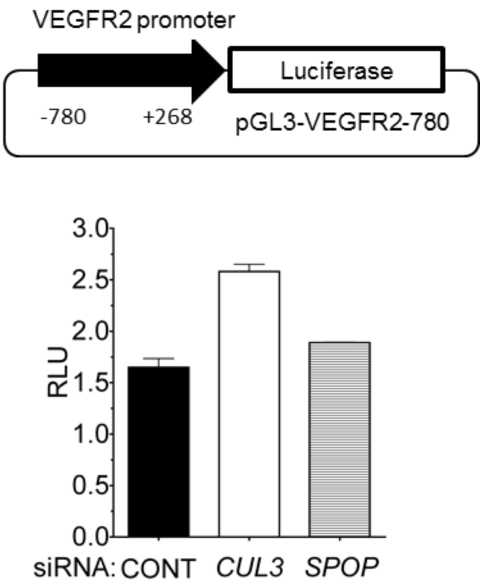

Supplementary Figure 3. (Higashiyama)

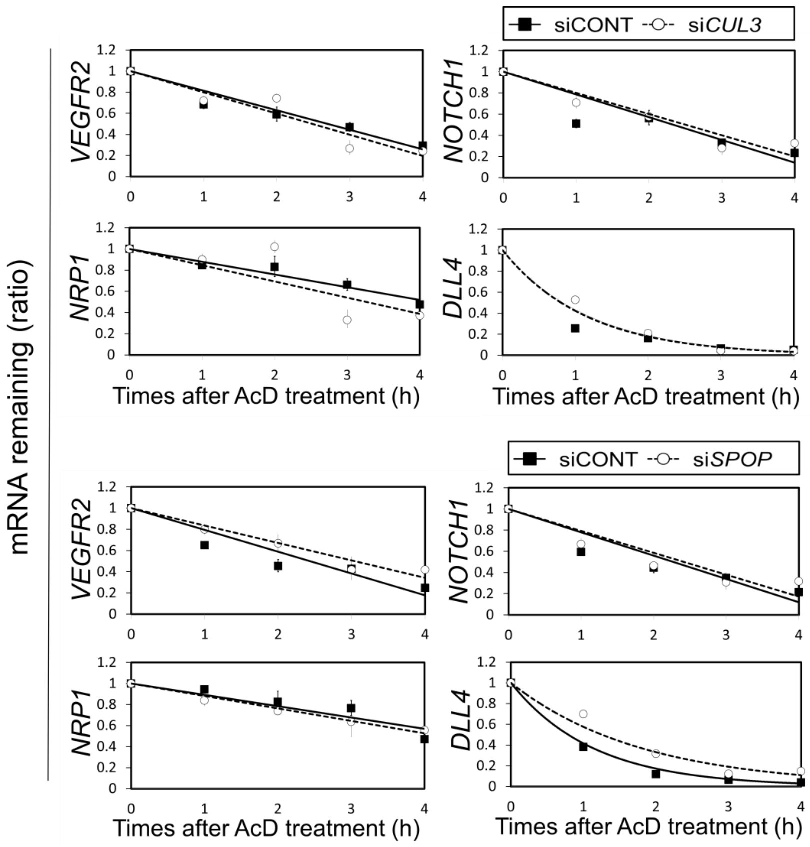

Supplementary Figure 4. (Higashiyama)

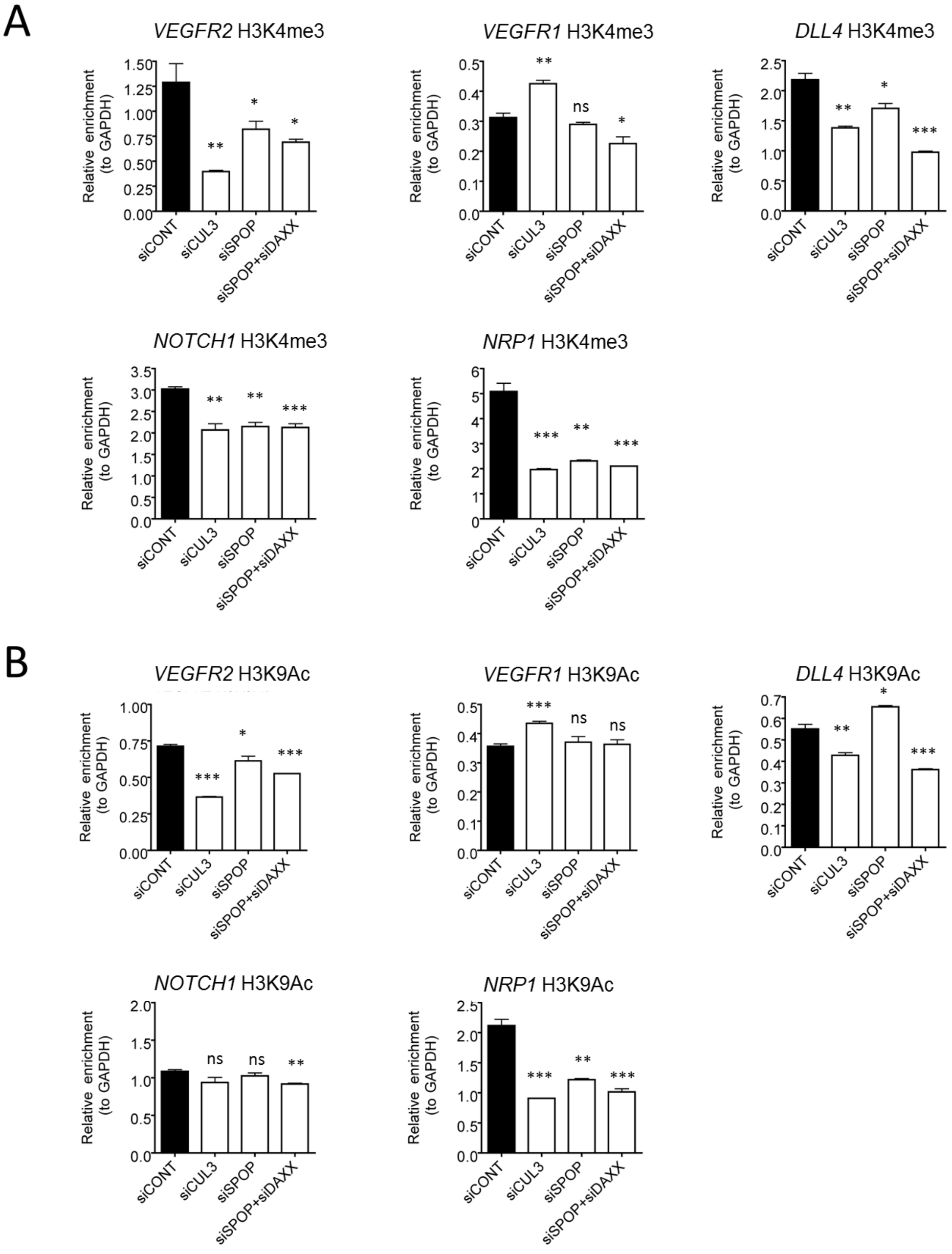

Supplementary Figure 5. (Higashiyama)

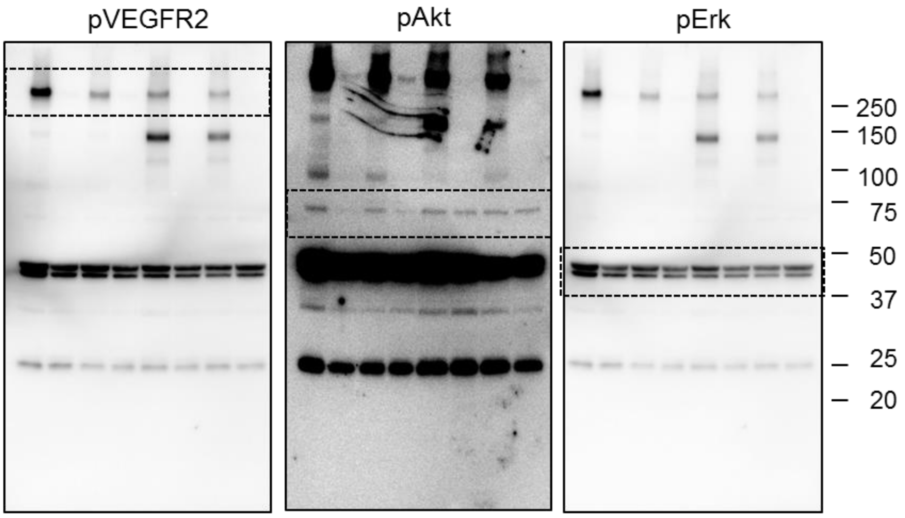

Fig. 1A; for phospho-VEGFR2, Akt and Erk (co-blot)

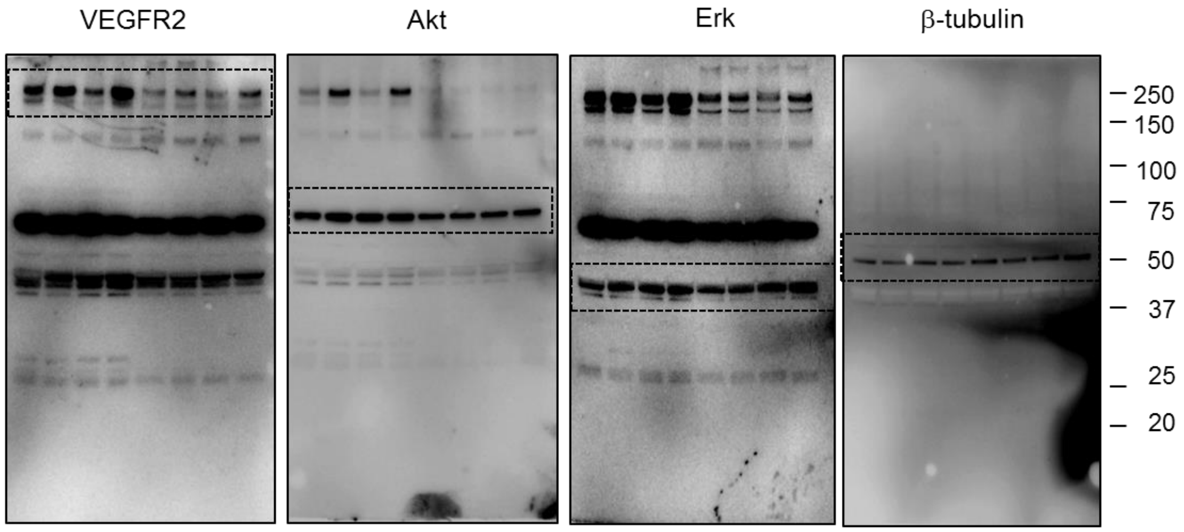

Fig. 1A; for VEGFR2, Akt and Erk (co-blot)

Fig. 1A; for β-tubulin

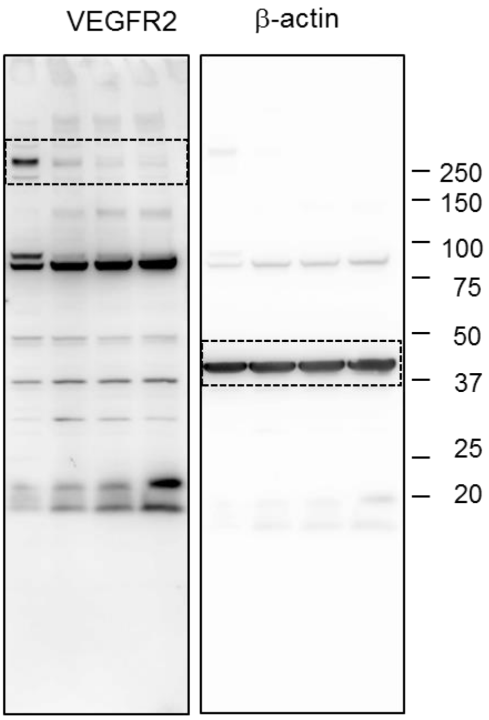

Fig. 1B; for VEGFR2 and β-actin (re-blot)

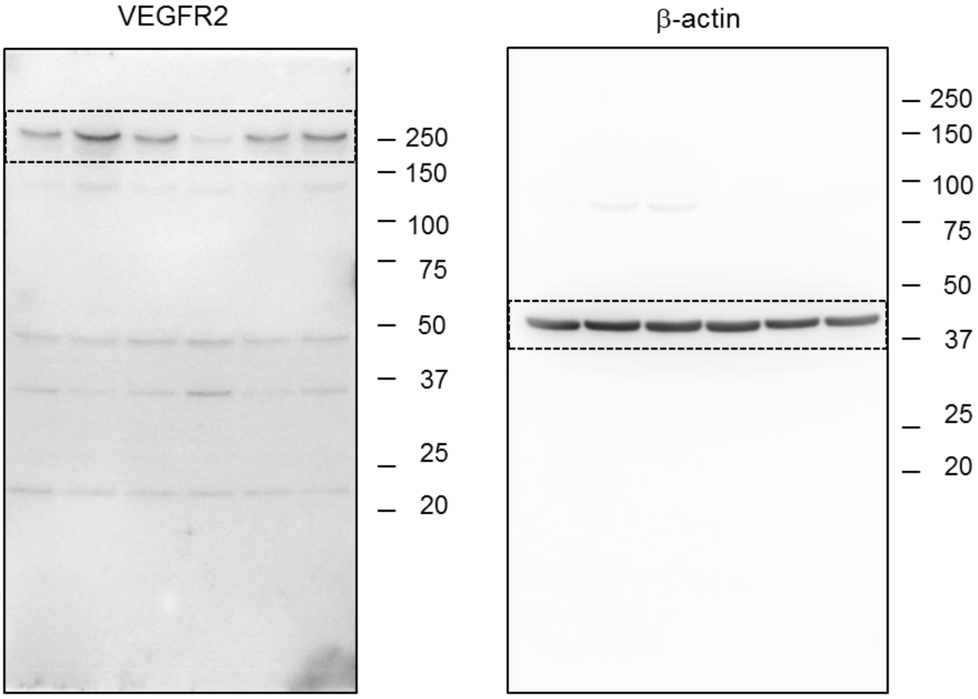

Fig. 2A; for VEGFR2 and β-actin

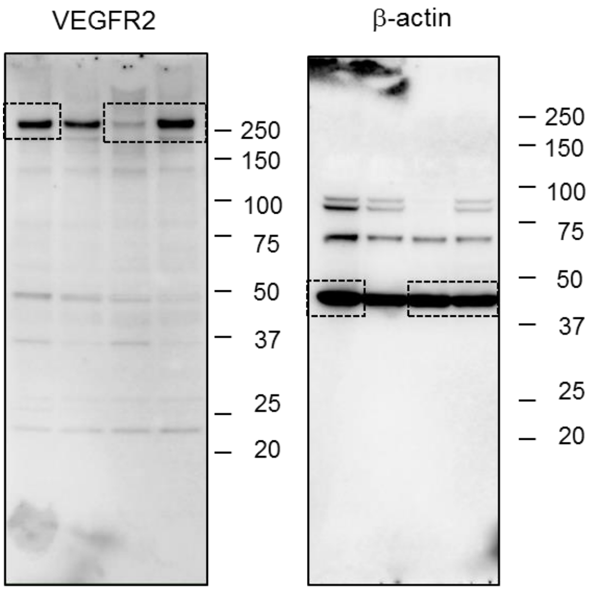

Fig. 2A right panel ; for VEGFR2 and β-actin

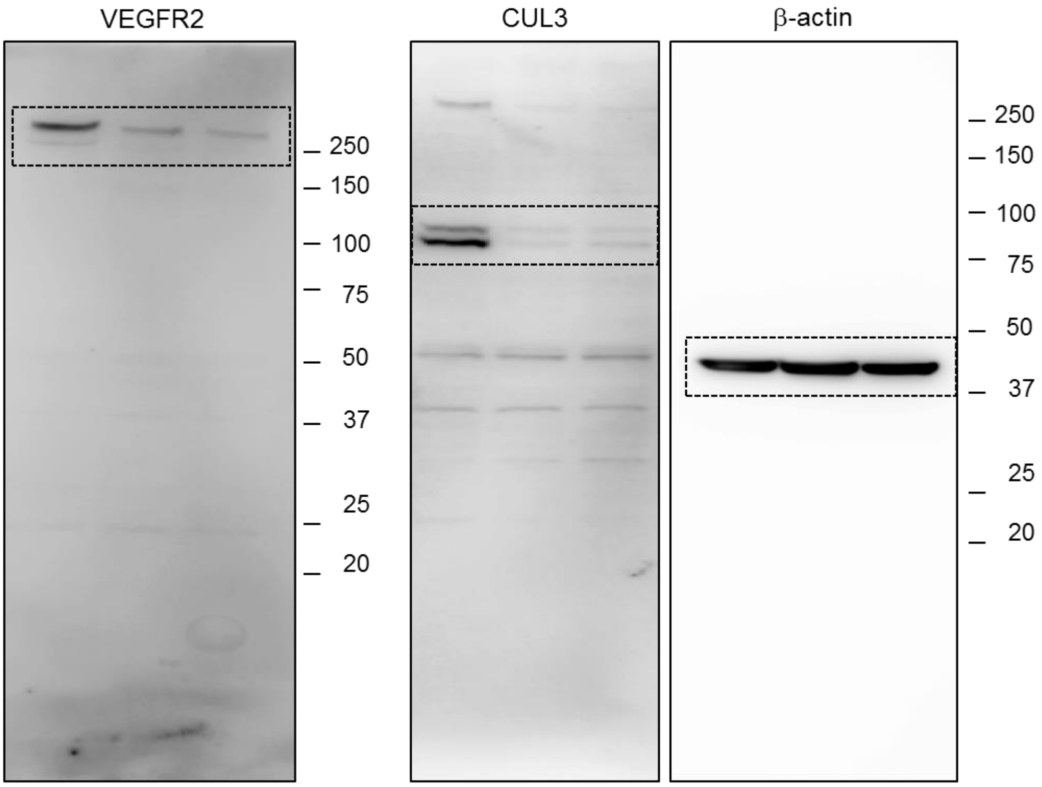

Fig. 2C; for VEGFR2, CUL3 and β-actin

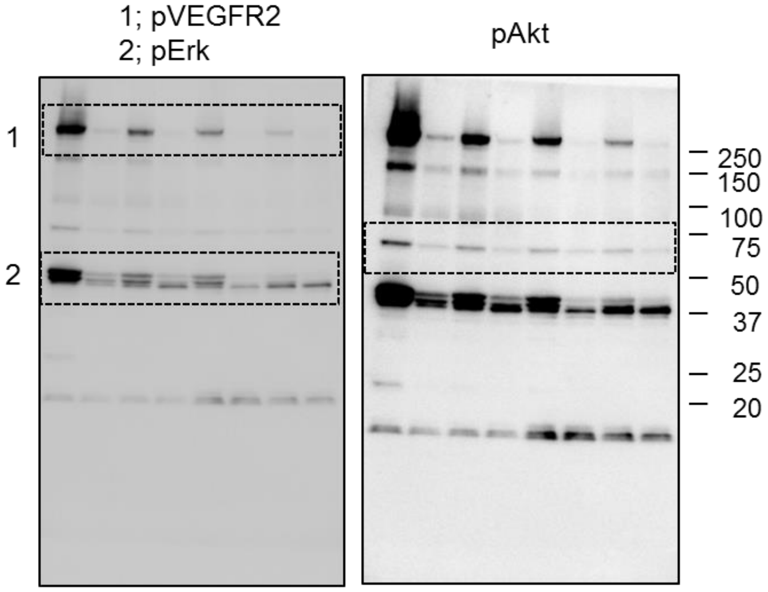

Fig. 3A; for phospho-VEGFR2, Akt and Erk (co-blot)

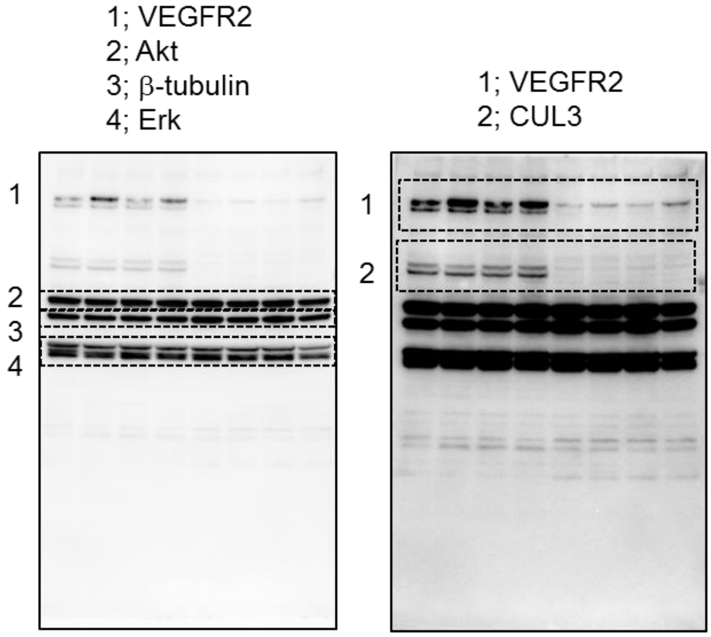

Fig. 3A; for VEGFR2, Akt, Erk, CUL3 and β-tubulin (co-blot)

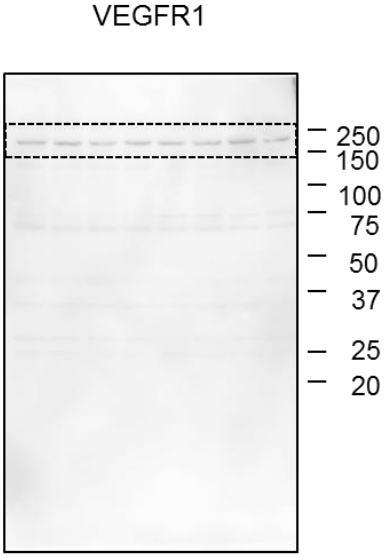

Fig. 3A; for VEGFR1

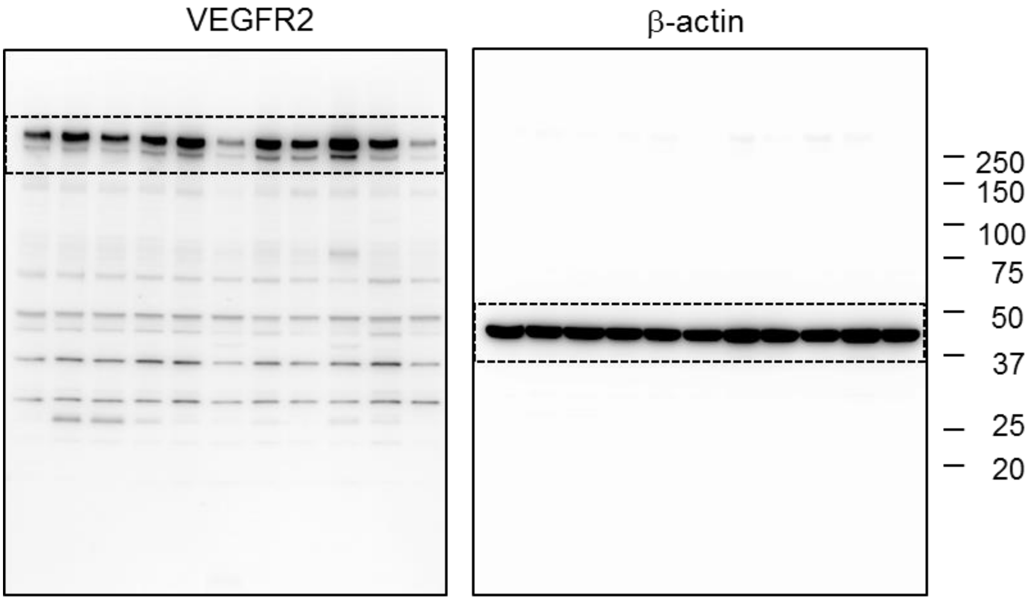

Fig. 4A; for VEGFR2 and β-actin (re-blot)

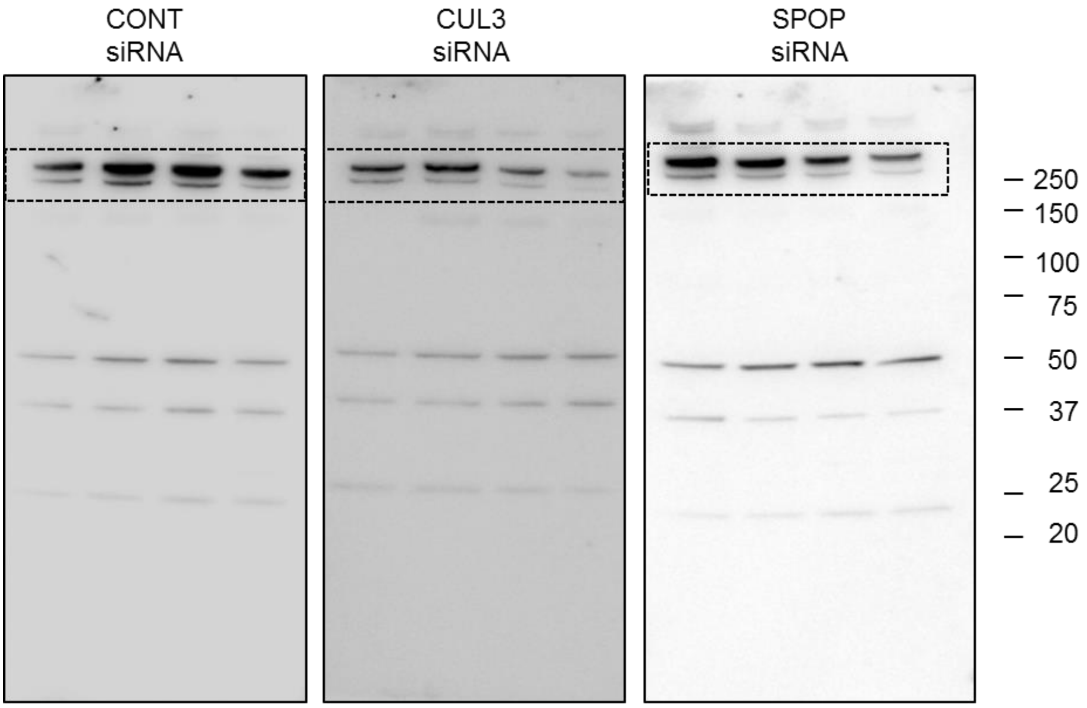

Fig. 4B; for VEGFR2

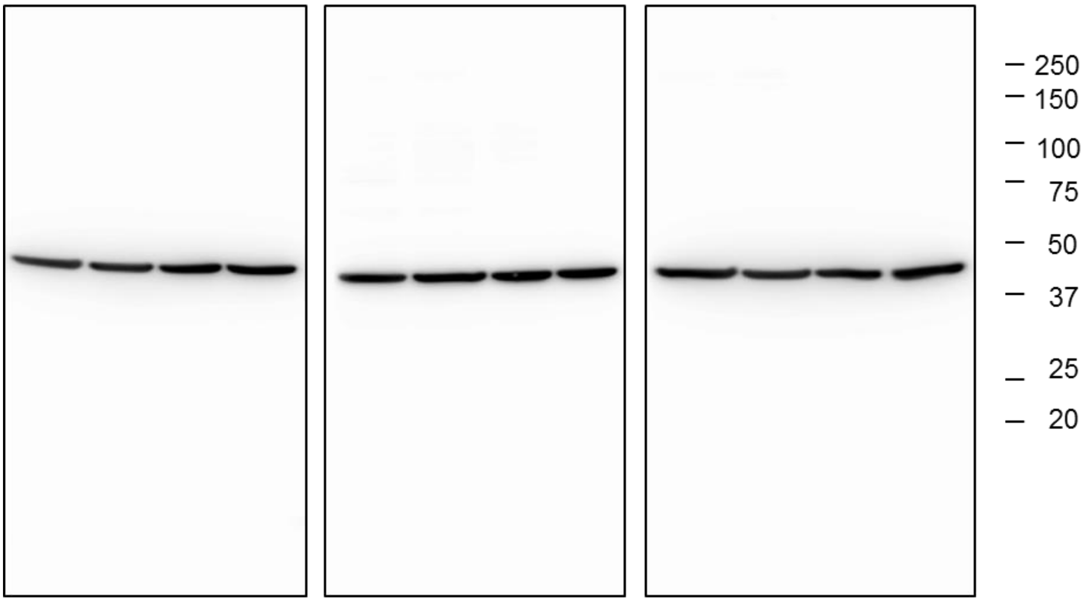

Fig. 4B; for β-actin (re-blot)

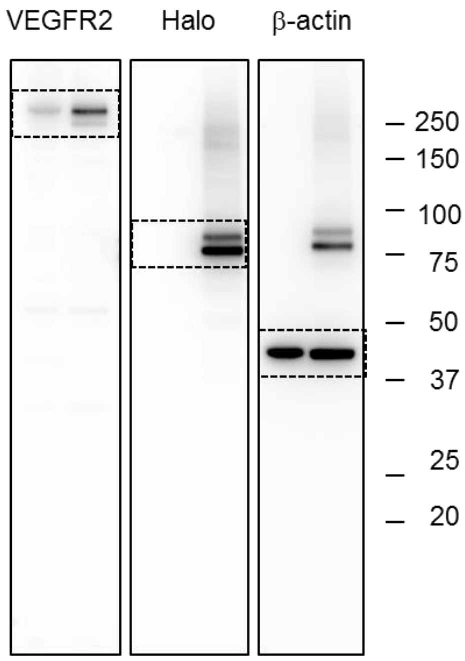

Fig. 4D; for VEGFR2, Halo and β-actin (re-blot)

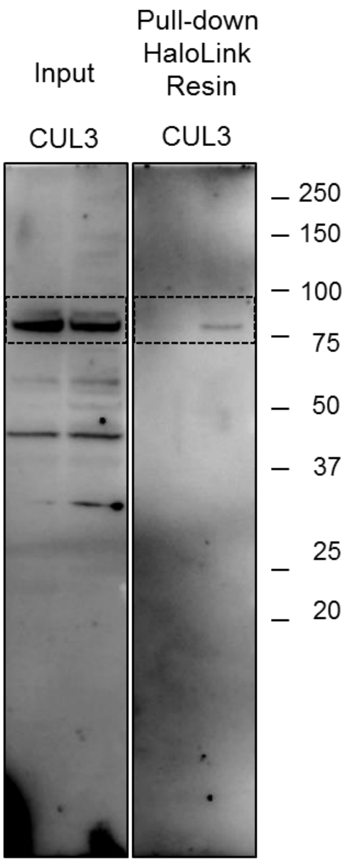

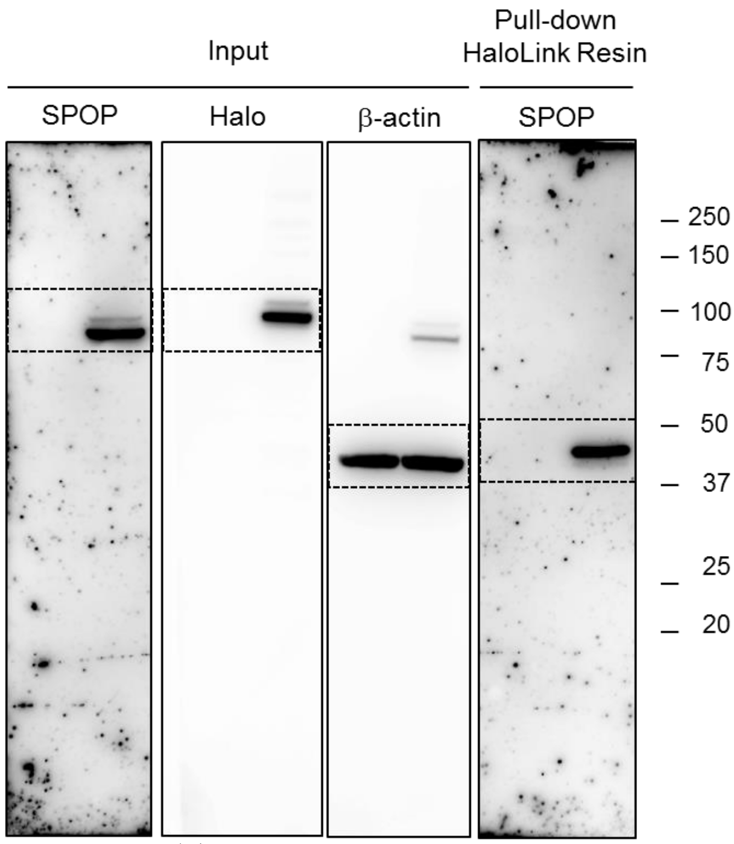

Fig. 4E; for CUL3

Fig. 4E; for VEGFR2, Halo, SPOP and β-actin (re-blot)

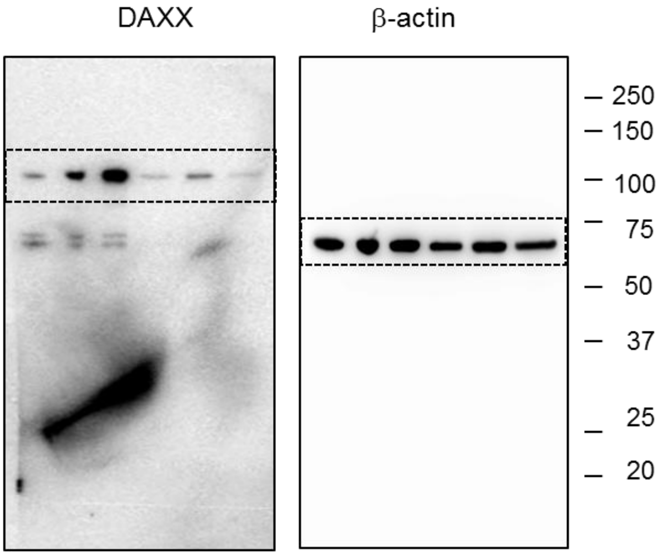

Fig. 5B; for DAXX and β-actin

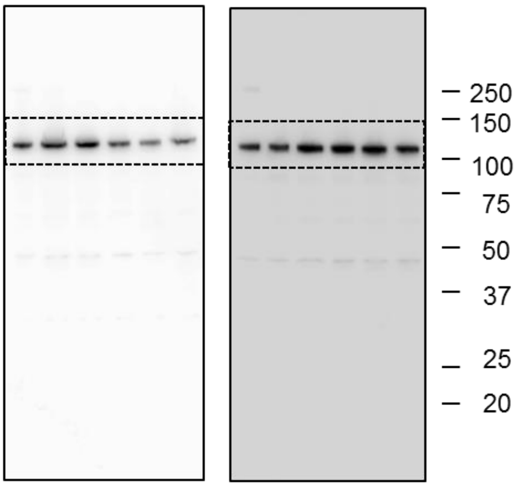

Fig. 5C; for DAXX

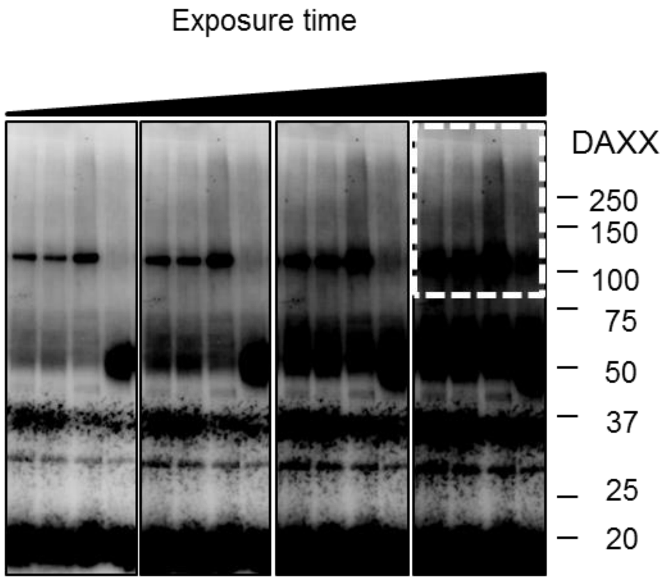

Fig. 5D; for DAXX

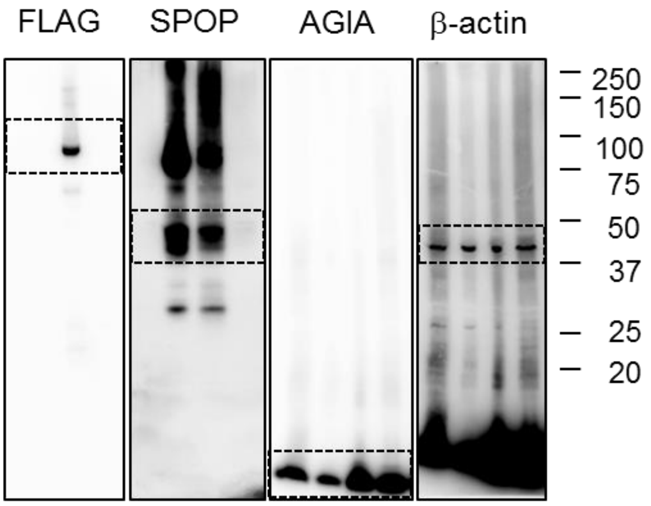

Fig. 5D; for FLAG, SPOP, AGIA and β-actin (re-blot)

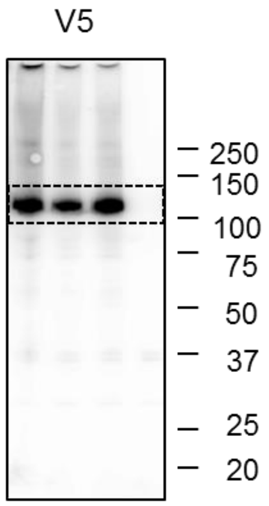

Fig. 5D; for V5

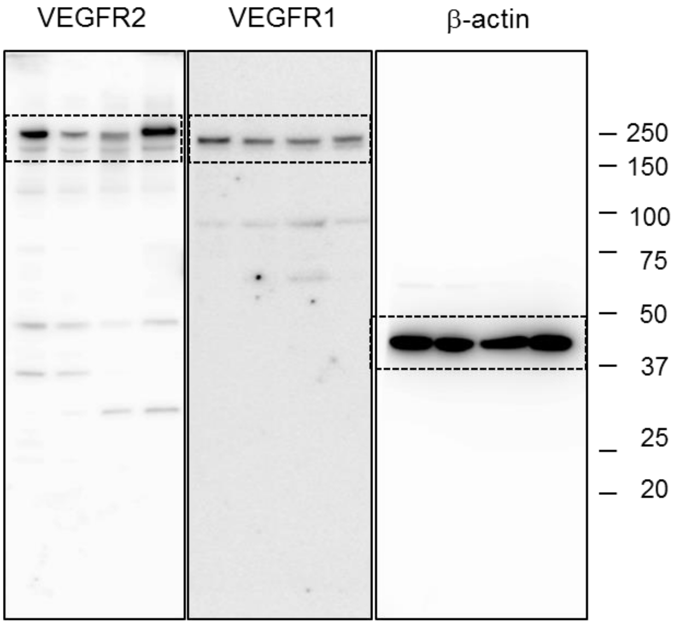

Fig. 5F; for VEGFR2, VEGFR1 and β-actin

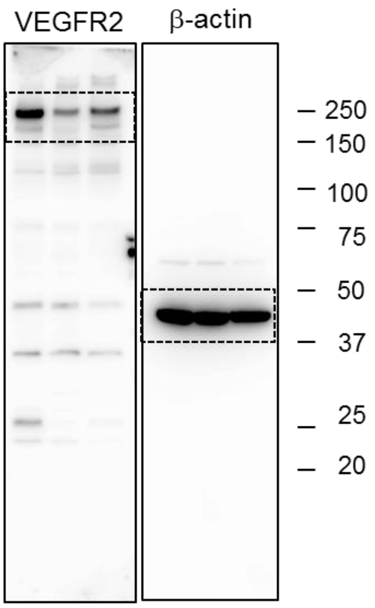

Fig. 5G; for VEGFR2 and β-actin

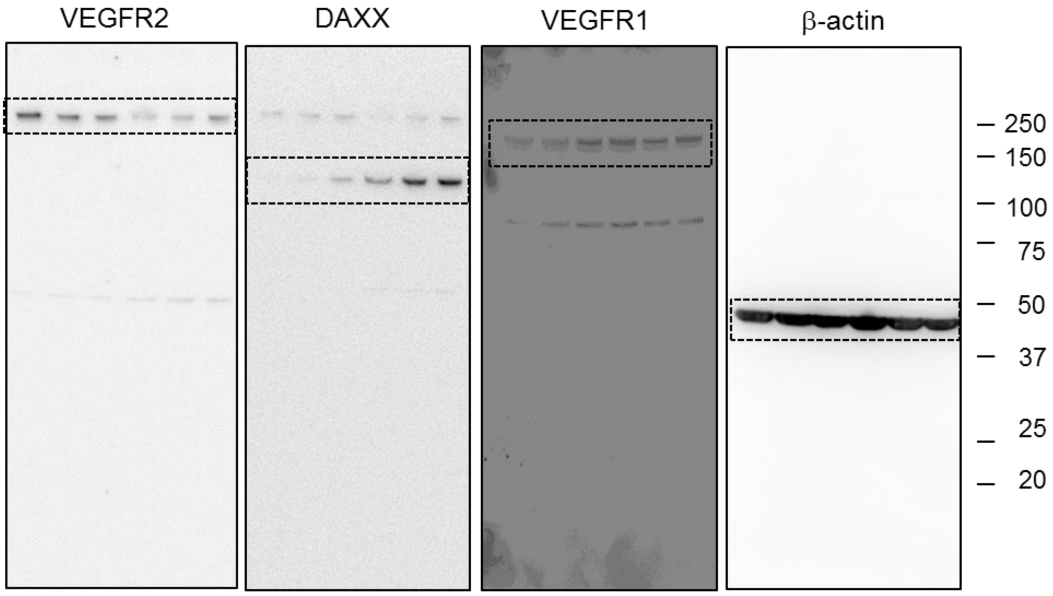

Fig. 5I; for VEGFR2, VEGFR1, DAXX and β-actin

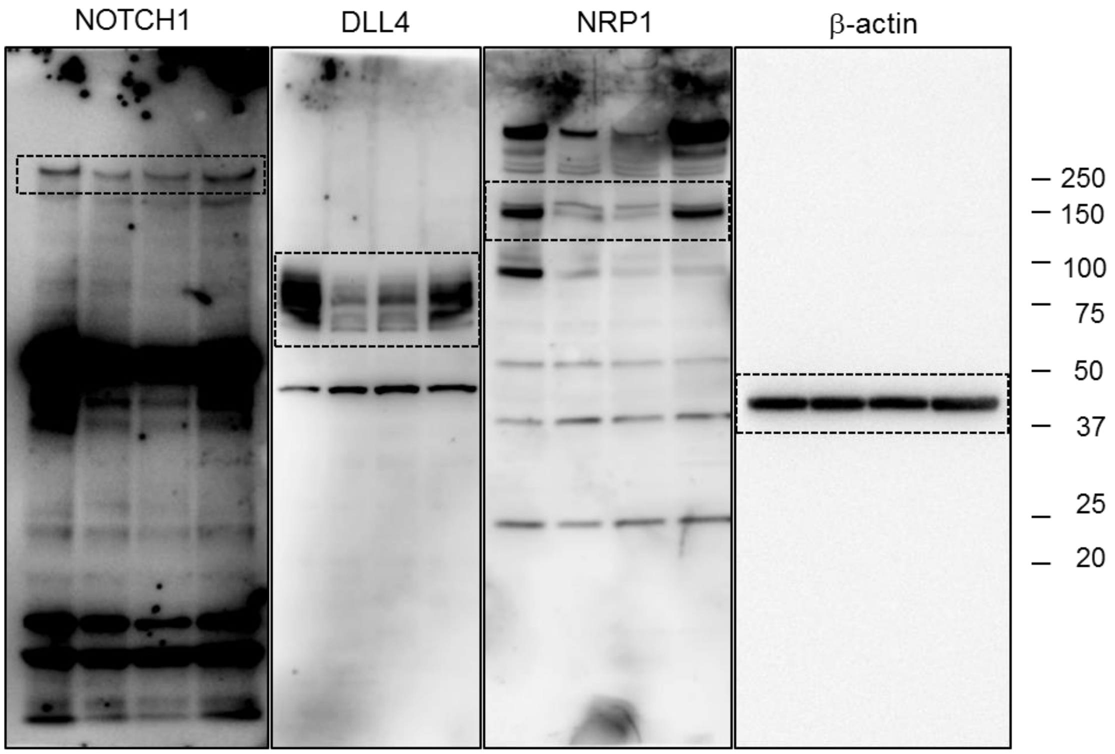

Fig. 6D; for NOTCH1, DLL4, NRP1 and β-actin

Supplementary Table 1. The list of siRNA IDs used in this study.

siRNA Name	Gene Name	siRNA ID
Mission siRNA (Sigma-Aldrich)	*CUL1*	SASI_Hs02_00335921
	CUL2	SASI_Hs01_00093148
	CUL3#1	SASI_Hs01_00102205
	CUL3#2	SASI_Hs01_00102207
	CUL4A	SASI_Hs01_00146692
	CUL5	SASI_Hs01_00112044
	KLHL5	SASI_Hs01_00157151
	KLHL9	SASI_Hs01_00222443
	KLHL20	SASI_Hs01_00123963
	KLHL24	SASI_Hs01_00163036
	RHOBTB1	SASI_Hs01_00138544
	SPOP	SASI_Hs01_00034100
	ZBTB33	SASI_Hs01_00199087
	KCTD20	SASI_Hs01_00237246
	TNFAIP1	SASI_Hs01_00131786
ON-TARGET plus Smart pool siRNA (Dharmacon)	*CUL3*	L-010224-00
	CUL4B	L-017965-00

Supplementary Table 2. Primer sequences for quantitative RT-PCR.

Target gene		Primer sequence 5'-3'
VEGFR2	forward	TGGGGGAGCGTGTCAGAAT
	reverse	CCGCTTTAATTGTGTGATTGGAC
VEGFR1	forward	TGGCTGCGACTCTCTTCTG
	reverse	CAAAGGAACTTCATCTGGGTCC
PTGS2	forward	TTGCTGGCAGGGTTGCTGGTGGTA
	reverse	CATCTGCCTGCTCTGGTCAATGGAA
VCAM1	forward	GCAAGGTTCCTAGCGTGTAC
	reverse	GGCTCAAGCTGTCATATTCAC
SPOP	forward	CCACAGTGCAGATCAGTTGAA
	reverse	CTCCAAGACATCCGAAGCAT
NOTCH1	forward	GAGGCGTGGCAGACTATGC
	reverse	CTTGTACTCCGTCAGCGTGA
DLL4	forward	TCCAACTGCCCTTCAATTTCAC
	reverse	CTGGATGGCGATCTTGCTGA
NRP1	forward	AAATGGGAATGGCTGATTCAG
	reverse	CTCCATCGAAGACTTCCACGTAGT
CUL3	forward	CGTAGACAGAGGCGCAATAAG
	reverse	GGCAGTGCATCACTCGTTCT
CUL4B	forward	AATGCGGAGCTGCTTTCAC
	reverse	CAGTGTCCTATGGTTGACTGC
β-actin	forward	CTGGAACGGTGAAGGTGACA
	reverse	AAGGGACTTCCTGTAACAATGCA
GAPDH	forward	TGCACCACCAACTGCTTAGC
	reverse	GGCATGGACTGTGGTCATGAG

Supplementary Table 3. Primer sequences for semi-quantitative RT-PCR.

Target gene		Primer sequence 5'-3'
CUL1	forward	CGCTGGCTTTGTGGCTGCTC
	reverse	TGTGGCGGCTGGCGTAGAA
CUL2	forward	CAGCGAAAGGGATGACAGAGAATG
	reverse	TGAGTAAGAGGCCACGCACCA
CUL3	forward	AGAGCGGAAAGGAGAAGTCGTAGA
	reverse	CTCAAAGTCACCCGCAATAGTT
CUL4A	forward	AACTCCACGCTGCCCTCCATCTG
	reverse	TGCTGCCCGCCCCTCACC
CUL5	forward	GAGTGGCTAAGAGAAGTTGGTATG
	reverse	TCTTCTCTCATCCTTTCTGTAGTG
